# Supplementary material for: The actin nucleation factors JMY and WHAMM enable a rapid Arp2/3 complex-mediated intrinsic pathway of apoptosis
Source: PLoS Genet. 2021 Apr 19;17(4):e1009512. doi: 10.1371/journal.pgen.1009512 (PMC8084344; doi:10.1371/journal.pgen.1009512)
Supplement: S1 Table — (PDF) [file pgen.1009512.s001.pdf]

S1 Table. Cell Lines

Parental Cells

Cell Line	Source
eHAP	Horizon Genomics (C669)
HAP1	Horizon Genomics (C631)
HeLa	UC Berkeley Cell Culture Facility
U2OS	UC Berkeley Cell Culture Facility

HAP1 Derivatives

KO Cell Line	Mutation	Predicted AAs	Source
Cortactin^{KO}	16bp deletion in exon 5 of 18	82/550, 49 post-shift	Horizon Genomics (HZGHC002628c003)
JMY$^{\text{KO-1A}}$	17bp deletion in exon 1 of 11	145/988, 4 post-shift	Horizon Genomics (HAP1_JMY_28380-03)
JMY$^{\text{KO-1B}}$	10bp deletion in exon 1 of 11	148/988, 30 post-shift	Horizon Genomics (HZGHC002630c002)
JMY$^{\text{KO-2}}$	2bp deletion in exon 2 of 11	362/988, 3 post-shift	Horizon Genomics (HZGHC002631c007)
N-WASP^{KO}	14bp deletion in exon 2 of 11	51/505, 12 post-shift	Horizon Genomics (HZGHC002632c003)
WASH Complex^{KO} (CCDC53^{KO})	1bp insertion in exon 3 of 7	56/194, 6 post-shift	Horizon Genomics (HZGHC004026c001)
WAVE1^{KO}	11bp deletion in exon 5 of 11	83/559, 41 post-shift	Horizon Genomics (HZGHC0033300c010)
WAVE2^{KO}	46bp deletion in exon 3 of 9	64/498, 41 post-shift	Horizon Genomics (HZGHC003327c004)
WAVE3^{KO}	2bp deletion in exon 4 of 10	82/502, 35 post-shift	Horizon Genomics (HZGHC003325c004)
WAVE Complex^{KO} (BRK1^{KO})	115bp insertion in exon 2 of 3	57/75, 45 post-shift	Horizon Genomics (HZGHC004027c003)

eHAP Derivatives

KO Cell Line		Mutation	Predicted AAs	Source
RhoD$^{\text{KO-2A}}$		22bp deletion in exon 2 of 5	44/210, 8 post-shift	Horizon Genomics (HZGHC005085c003)
RhoD$^{\text{KO-2B}}$		1bp deletion in exon 2 of 5	48/210, 11 post-shift	Horizon Genomics (HZGHC005085c011)
WHAMM$^{\text{KO-2}}$		10bp deletion in exon 2 of 10	204/809, 7 post-shift	Horizon Genomics Mathiowetz et al., (2017)
WHAMM$^{\text{KO-4}}$		7bp deletion in exon 4 of 10	321/809, 17 post-shift	Horizon Genomics (HZGHC001060c001)
WHAMM/JMY$^{\text{DKO-1}}$	WHAMM:	10bp deletion in exon 2 of 10	204/809, 7 post-shift	Horizon Genomics (HZGHC004884C012)
	JMY:	16bp deletion in exon 1 of 11	112/998, 64 post-shift	
WHAMM/JMY$^{\text{DKO-2}}$	WHAMM:	10bp deletion in exon 2 of 10	204/809, 7 post-shift	Horizon Genomics (HZGHC004884C007)
	JMY:	35bp deletion in exon 2 of 11	362/998, 33 post-shift	
