# Supplementary material for: The actin nucleation factors JMY and WHAMM enable a rapid Arp2/3 complex-mediated intrinsic pathway of apoptosis
Source: PLoS Genet. 2021 Apr 19;17(4):e1009512. doi: 10.1371/journal.pgen.1009512 (PMC8084344; doi:10.1371/journal.pgen.1009512)
Supplement: S2 Table — (PDF) [file pgen.1009512.s002.pdf]

S2 Table. RNA and DNA

siRNAs

Target	Identifier		
Control (Fig 4, 5, 7, 8, S5, S6, S11)	Sigma SIC001		
Arp2/3 Complex (Fig 8)	ARP3: Ambion 138029	ARPC4: Ambion 214781	
JMY (Fig 4, 5, S5)	A: SASI_HS01_00366091	B: SASI_HS01_00081989	
TP53 (Fig 5, S6)	A: SASI_HS02_00302766	B: SASI_HS02_00302767	C: SASI_HS02_00302768
RHOD (Fig 7, S11)	A: SASI_HS01_00186024	B: SASI_HS01_00186023	
WHAMM (Fig S5)	A: Invitrogen HSS151263	B: Invitrogen HSS151264	

RT-PCR Primers

Target	Sequence	
β-ACTIN (Fig 6, 7, S11)	F	GCTCGTCGTCGACAACGGCT
	R	GGTCATCTTCTCGCGGTTGG
BAX (Fig 6)	F	GTCTTTTTCCGAGTGGCAGC
	R	GGAGACAGGGACATCAGTCG
BBC3 (Fig 6)	F	CTGCCAGATTTGTGGTCCTCAG
	R	AATTGGGCTCCATCTCGGGG
BCL2 (Fig 6)	F	GAACTGGGGGAGGATTGTGG
	R	CAGAGACAGCCAGGAGAAATC
CDKN1A (Fig S8)	F	GGCCCGTGAGCGATGGAACT
	R	GGAGTGGTAGAAATCTGTCATGCTGG
GAPDH (Fig 6, 7, S5, S8, S11)	F	CCTCCTGCACCACCAACTGC
	R	CTCCGACGCCTGCTTCACCA
RAB1A (Fig 6)	F	GTTATGCCAGTGAAAATGTCAACAAATTGT
	R	TGCTCTGAATTTTAACATTGGACTTCTCAG
RAB1B (Fig 6)	F	GCGAGAACGTCAATAAGCTCC
	R	GTCGATCTTGAGATTGGGCCGCT
RHOD (Fig 6, 7, S11)	F (nt 225-247)	AGATGACTATGACCGCCTGCGGC
	F (nt 124-147)	TTCCCCGAGAGCTACACCCCCACG
	R	ACGGCGTGGACGTTGTCATGGAG
WHAMM (Fig 6, S5)	F	CTCCGTGCTCTGTCCTCATCCTCTCA
	R	CTAACCATCCCACTGGCCAGGGTCCT

Cloning Primers

Description	Species	Sequence	
JMY-Start	Mouse	F	ATCATCAGATCTATGTCGTTCGCGCTGGAGGA
JMY-Stop	Mouse	R	ATCATCGCGGCCGCTTACTAGTTCTCCCAGTCTGTGCAC
JMY-ΔCA	Mouse	R	ATCATCGCGGCCGCTTAGGATTCTCTCAAAACTTCCTTCTG
JMY-ΔWWW	Mouse	F	ATCATCACCGGTCAGAAGGAAGTTTTGAGAGAATCC
		R	ATCATCACCGGTGTGTGCTGCGGGGGCACRGGC
RhoD-DN	Mouse	F	TGCGGGAAGAACTCACTGATGATGGTC
		R	GACCATCATCAGTGAGTTCTTCCCGCA

Plasmids

Description	Vector	Species	AA	R.E. Sites	Source
pGFP (pKC425) (vector)	pCDNA3::GFP	N/A	N/A	N/A	Campellone et al., 2008
pGFP-JMY	pCDNA3::GFP	Mouse	1-983	BamH1-Not1	This Study
pGFP-JMY(ΔCA)	pCDNA3::GFP	Mouse	1-941	BamH1-Not1	This Study
pGFP-JMY(ΔWWW)	pCDNA3::GFP	Mouse	1-856/934-983	BamH1-Not1	This Study
pKC-LAP-C1 (vector)	pKC-LAP-C1	N/A	N/A	N/A	Campellone et al., 2008
pKC-LAP-RhoD	pKC-LAP-C1	Mouse	1-210	BglII-EcoR1	This Study
pKC-LAP-RhoD(DN)	pKC-LAP-C1	Mouse	1-210 (T31N)	BglII-EcoR1	This Study
