# Supplementary material for: The actin nucleation factors JMY and WHAMM enable a rapid Arp2/3 complex-mediated intrinsic pathway of apoptosis
Source: PLoS Genet. 2021 Apr 19;17(4):e1009512. doi: 10.1371/journal.pgen.1009512 (PMC8084344; doi:10.1371/journal.pgen.1009512)
Supplement: S3 Table — (PDF) [file pgen.1009512.s003.pdf]

S3 Table. Immunofluorescence and Immunoblotting Reagents

Target	Probe		Conc.	Identifier
Primary Antibodies (Immunofluorescence)				
Actin (Fig S12)	anti-Beta-Actin	Mouse	1:1,000	Proteintech (66009-1-Ig)
Casp-3^{Cleaved} (Fig 3, 4, 7, 8, S11)	anti-Cleaved Caspase-3	Rabbit	1:1,000	Cell Signaling Technology (9664)
Cytochrome *c* (Fig 2, 7, 9, 10, S11, S12)	anti-Cytochrome *c*	Mouse	1:500	Cell Signaling Technology (12963)
γH2AX (Fig S4)	anti-Phospho-Histone H2A.X	Rabbit	1:1,000	Cell Signaling Technology (9718)
Mitochondria (Fig 2, 7, S11)	anti-AIF	Rabbit	1:1,000	Cell Signaling Technology (5318)
JMY (Fig 9, 10, S12)	anti-JMY	Rabbit	1:1,000	Proteintech (25098-1-AP)
p53 (Fig S7)	anti-p53	Rabbit	1:1,000	Proteintech (10442-1-AP)
p21 (Fig S8)	anti-p21	Rabbit	1:250	Cell Signaling Technology (2947)
Primary Antibodies (Immunoblotting)				
Actin (Fig 8, S1)	anti-Beta-Actin	Mouse	1:10,000	Proteintech (66009-1-Ig)
ARP3 (Fig 8)	anti-ARP3	Mouse	1:1,000	Sigma (A5979)
ARPC2 (Fig 8)	anti-p34-Arc/ARPC2	Rabbit	1:1,000	EMD Millipore (07-227-I)
Casp-9^{Pro} & Casp-9^{Cleaved} (Fig 3, S11)	anti-Total Caspase-9	Rabbit	1:500	Cell Signaling Technology (9502)
Casp-3^{Pro} & Casp-3^{Cleaved} (Fig 3)	anti-Total Caspase-3	Mouse	1:500	Cell Signaling Technology (9668)
Casp-3^{Cleaved} (Fig 8, S11)	anti-Cleaved Caspase-3	Rabbit	1:250	Cell Signaling Technology (9664)
Cortactin (Fig S1)	anti-Cortactin	Rabbit	1:2,000	EMD Millipore (05-180)
γH2AX (Fig S4)	anti-Phospho-Histone H2A.X	Rabbit	1:1,000	Cell Signaling Technology (9718)
GAPDH (Fig 4, 5, 8, S1, S7)	anti-GAPDH	Mouse	1:10,000	Proteintech (60004-1-Ig)
JMY (Fig 1)	anti-JMY	Goat	1:350	Santa Cruz Biotech (sc-10027)
JMY (Fig 4, 5, 8, S5)	anti-JMY	Rabbit	1:1,000	Proteintech (25098-1-AP)
N-WASP (Fig S1)	anti-N-WASP	Guinea Pig	1:1,000	Duleh et al., 2010
p53 (Fig 5, S6, S7)	anti-p53	Rabbit	1:250	Proteintech (10442-1-AP)
p53$^{phospho-S15}$ (Fig S7)	anti-Phospho-p53 (S15)	Rabbit	1:500	Cell Signaling Technology (9284)
p53$^{phospho-S46}$ (Fig S7)	anti-Phospho-p53 (S46)	Rabbit	1:500	Cell Signaling Technology (2521)
p53$^{acetyl-K382}$ (Fig S7)	anti-Acetyl-p53 (K382)	Rabbit	1:500	Cell Signaling Technology (2525)
Strumpellin (Fig S1)	anti-Strumpellin	Rabbit	1:1,000	Abcam (ab101222)
Tubulin (Fig 1, 3, 4, 5, 8, S1, S4, S5, S6, S7, S11)	anti-Beta-Tubulin	Mouse	1:10,000	DSHB (E7)
WASH (Fig S1)	anti-WASH	Rabbit	1:1,000	Duleh et al., 2010
WAVE1 (Fig S1)	anti-WAVE1	Rabbit	1:1,000	Abcam (ab50356)
WAVE2 (Fig S1)	anti-WAVE2	Rabbit	1:1,000	Cell Signaling Technology (3659)
WHAMM (Fig 1)	anti-WHAMM	Rabbit	1:1,000	Shen et al., 2012
Secondary Antibodies (Immunofluorescence)				
Mouse IgG	Alexa 350, 488, 555, 647 anti-mouse	Goat	4 μg/ml	Life Technologies (e.g. A11029)
Rabbit IgG	Alexa 488, 555, 647 anti-rabbit	Goat	4 μg/ml	Life Technologies (e.g. A11034)
Secondary Antibodies (Immunoblotting)				
Mouse IgG	HRP anti-Mouse	Sheep	1:10,000	GE Healthcare (NXA931)
Rabbit IgG	HRP anti-Rabbit	Donkey	1:10,000	GE Healthcare (NA934V)
Goat IgG	HRP anti-Goat	Donkey	1:5,000	Jackson ImmunoRes. (705-035-147)
Mouse IgG	IRDye 680, 800 anti-Mouse	Donkey	0.05 μg/ml	LI-COR (e.g. 926-32212)
Rabbit IgG	IRDye 680, 800 anti-Rabbit	Donkey	0.05 μg/ml	LI-COR (e.g. 926-32213)

Molecular Probes (Fluorescence)

Target	Probe	Conc.	Identifier
Active Caspase-3/7 (Fig 3, 7, 9, S11)	Caspase-3/7 Green Detection Reagent	5 μM	Invitrogen (C10423)
DNA (Fig 1, 3, 4, 5, 7, 8, S1, S2, S5, S6, S8, S11)	Hoechst 33342 Solution	2 μg/ml	Thermo Scientific (62249)
DNA (Fig 2, 3, 4, 7, 8, 9, 10, S1, S4, S7, S8, S11, S12)	4′,6-diamidino-2-phenylindole (DAPI)	1 μg/ml	Invitrogen (D1306)
F-actin (Fig 9, 10, S1, S12)	Alexa488-Phalloidin	0.2 U/ml	Invitrogen (A12379)
F-actin (Fig S4)	Alexa555-Phalloidin	0.4 U/ml	Invitrogen (A34055)
F-actin (Fig 8, 10)	Alexa647-Phalloidin	0.4 U/ml	Invitrogen (A22287)
Mitochondria (Fig 9, S12)	MitoTracker Red CM-H_2XRos	500 nM	Invitrogen (M7513)
Nucleic Acids (Fig 1, 4, 5, 7, 8, S1, S2, S5, S6, S8, S11)	Propidium Iodide	2 μg/ml	Invitrogen (P3566)
Phosphatidylserine (Fig 1, 4, 5, 7, 8, S1, S2, S5, S6, S8, S11)	Alexa488-Annexin V	8 μM	Invitrogen (A13201)
