# Supplementary figures and images for: The actin nucleation factors JMY and WHAMM enable a rapid Arp2/3 complex-mediated intrinsic pathway of apoptosis
Source: PLoS Genet. 2021 Apr 19;17(4):e1009512. doi: 10.1371/journal.pgen.1009512 (PMC8084344; doi:10.1371/journal.pgen.1009512)

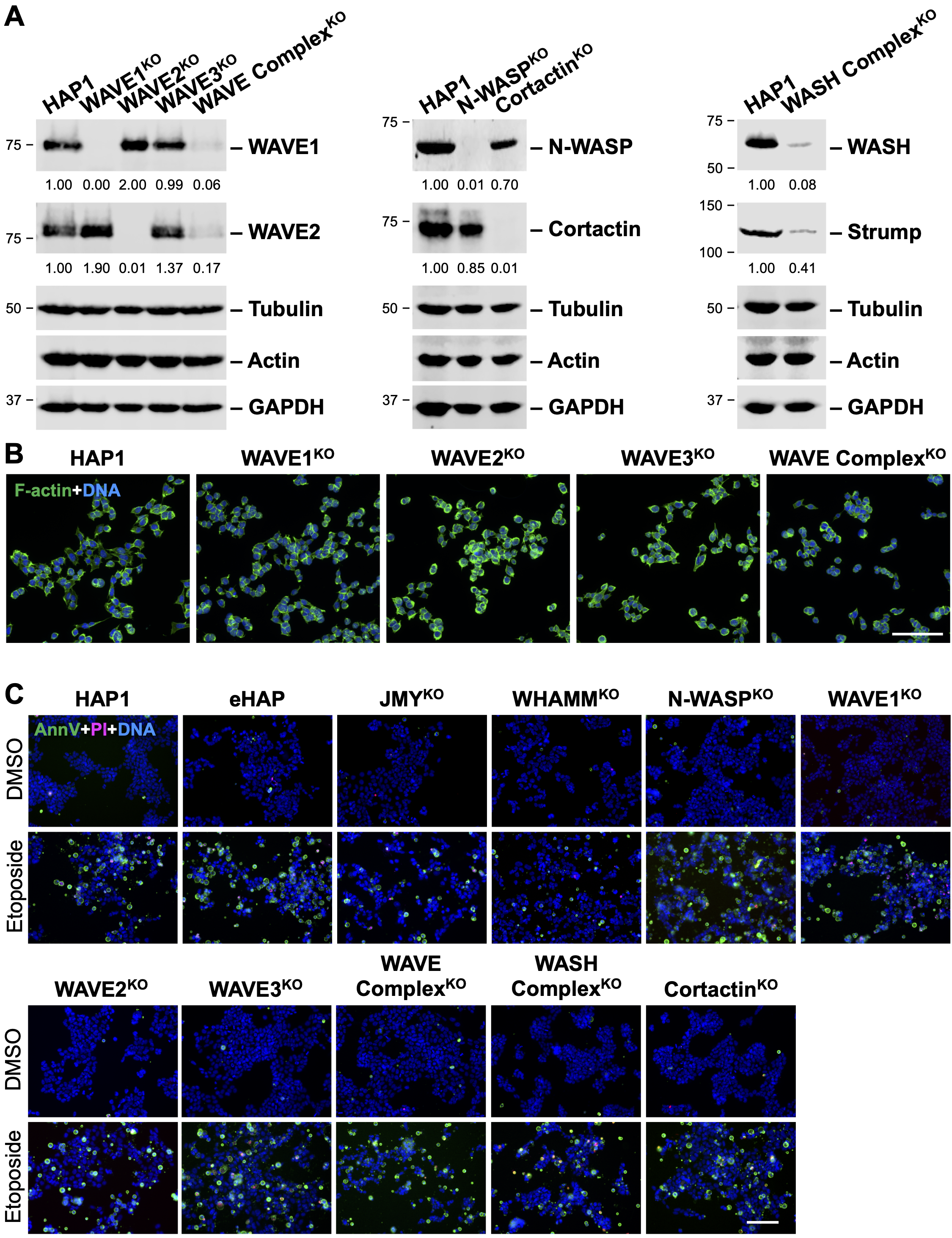

Supplement: S1 Fig — (A) HAP1, WAVE1KO, WAVE2KO, WAVE3KO, WAVE ComplexKO (BRK1KO), N-WASPKO, CortactinKO, and WASH ComplexKO (CCDC53KO) cells were collected, lysed, and immunoblotted with different combinations of antibodies to WAVE1, WAVE2, N-WASP, Cortactin, WASH, and Strumpellin. Tubulin, actin, and GAPDH were used as loading controls. The relative quantities of WASP-family proteins in each cell line (shown beneath their respective blots) were determined by densitometry and normalization to the loading controls in these representative experiments confirming the WASP-family member deficiencies. (B) HAP1, WAVE1KO, WAVE2KO, WAVE3KO, and WAVE ComplexKO cells were fixed and stained with phalloidin (F-actin; green) and DAPI (DNA; blue). Scale bar, 100μm. Note the rounder morphologies of the WAVE ComplexKO cells compared to the other cells. (C) Parental (HAP1, eHAP) and WASP-family knockout (JMYKO-1A, WHAMMKO-2, N-WASPKO, WAVE1KO, WAVE2KO, WAVE3KO, WAVE ComplexKO, WASH ComplexKO, CortactinKO) cells were treated with DMSO or 5μM etoposide for 6h and stained with Alexa488-AnnexinV (AnnV; green), Propidium Iodide (PI; magenta), and Hoechst (DNA; blue). Scale bar, 100μm. These panels depict some of the imaging data that was incorporated into the summary graph in Fig 1. (TIF) [file pgen.1009512.s004.tif]

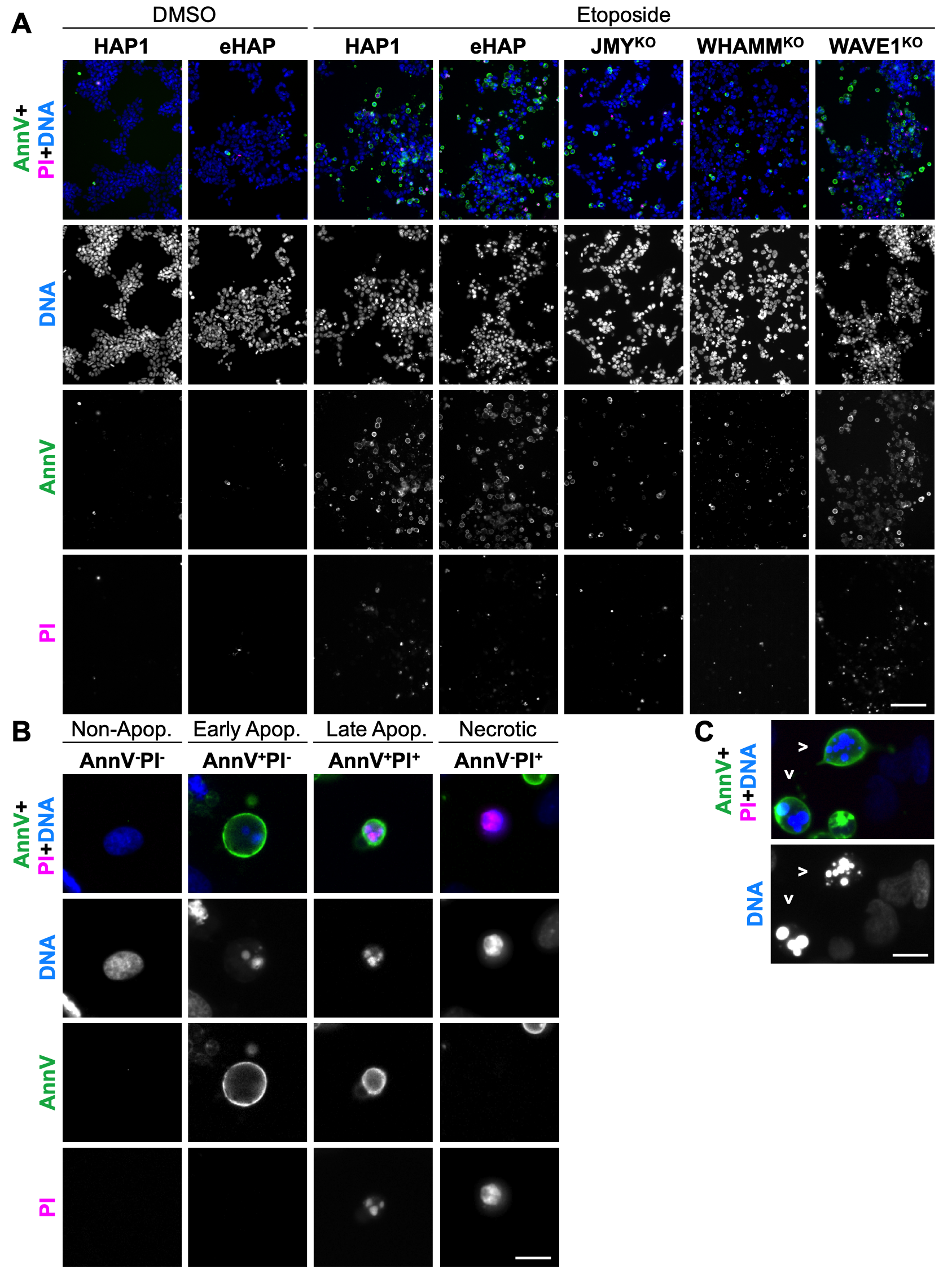

Supplement: S2 Fig — (A) Parental (HAP1, eHAP) and WASP-family knockout (JMYKO-1A, WHAMMKO-2, WAVE1KO) cells were treated with DMSO or 5μM etoposide for 6h and stained with Alexa488-AnnV (green), PI (magenta), and Hoechst (DNA; blue). Scale bar, 100μm. These panels display the individual grayscale channels that comprise the merged images in Fig 1. (B-C) Representative examples of AnnV-negative/PI-negative (AnnV-PI-) non-apoptotic, AnnV-positive/PI-negative (AnnV+PI-) early apoptotic, AnnV/PI double-positive (AnnV+PI+) late apoptotic, or AnnV-negative/PI-positive (AnnV-PI+) necrotic cells are shown. Arrowheads in C highlight examples of hoechst-stained DNA condensation and nuclear fragmentation in HAP1 cells. Scale bars, 25μm. (TIF) [file pgen.1009512.s005.tif]

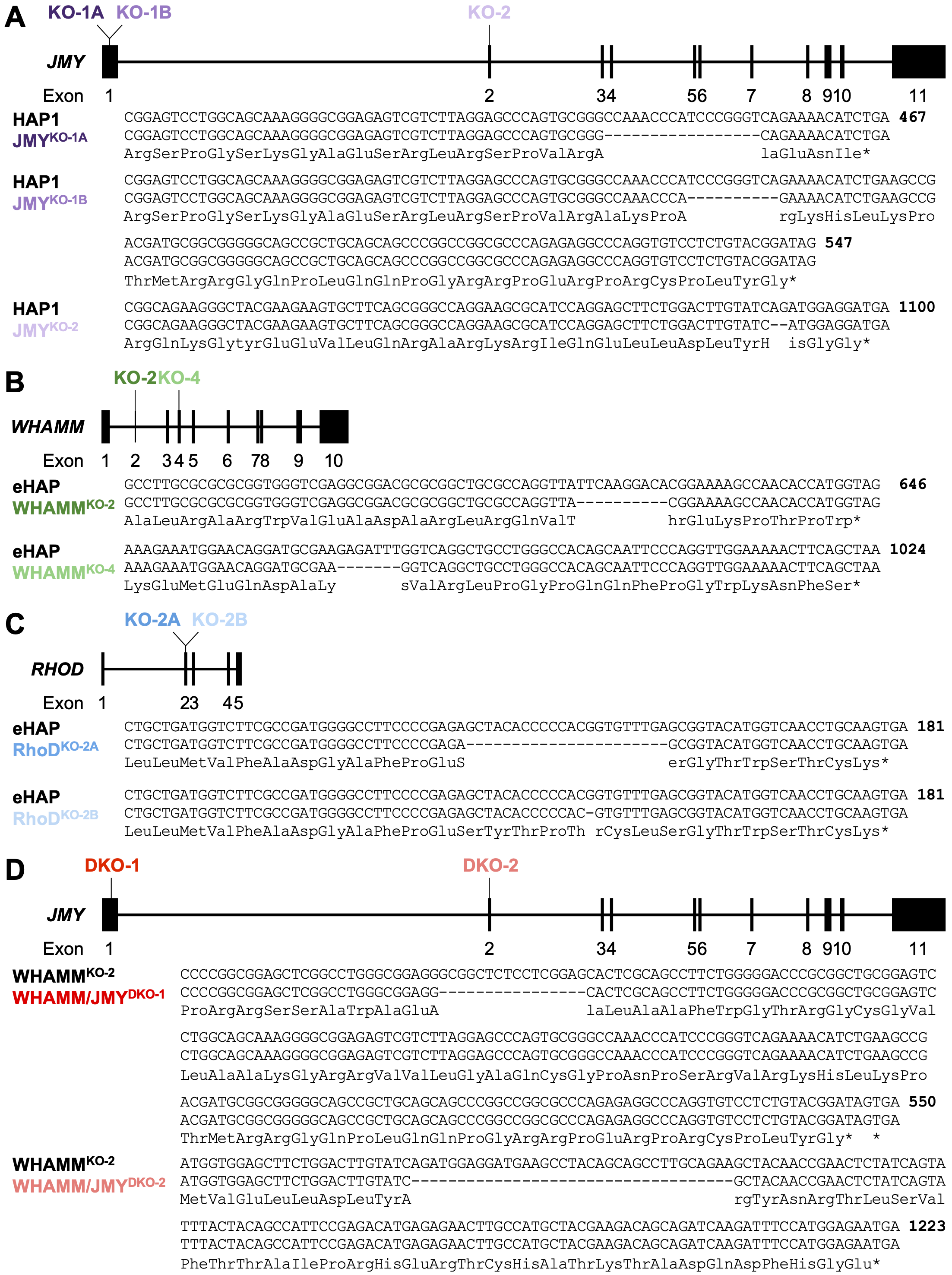

Supplement: S3 Fig — (A) HAP1 cells were CRISPR/Cas9 engineered using guide RNAs to the first or second exon of the JMY gene. A 17bp deletion in JMYKO-1A, a 10bp deletion in JMYKO-1B, and a 2bp deletion in JMYKO-2 resulted in frameshifts and premature stop codons. (B) eHAP cells were treated with guide RNAs to the second or fourth exon of the WHAMM gene. A 10bp deletion in WHAMMKO-2 and a 7bp deletion in WHAMMKO-4 resulted in frameshifts and premature stop codons. (C) eHAP cells were treated with guide RNAs to the second exon of the RHOD gene. A 22bp deletion in RhoDKO-2A is predicted to result in defective splicing (not shown) and/or a frameshift (shown), while a 1bp deletion in RhoDKO-2B results in a simple frameshift and premature stop codon. (D) WHAMMKO-2 cells were treated with guide RNAs to the first or second exon of the JMY gene. A 16bp deletion in WHAMM/JMYDKO-1 and a 35bp deletion in WHAMM/JMYDKO-2 resulted in frameshifts and premature stop codons. (TIF) [file pgen.1009512.s006.tif]

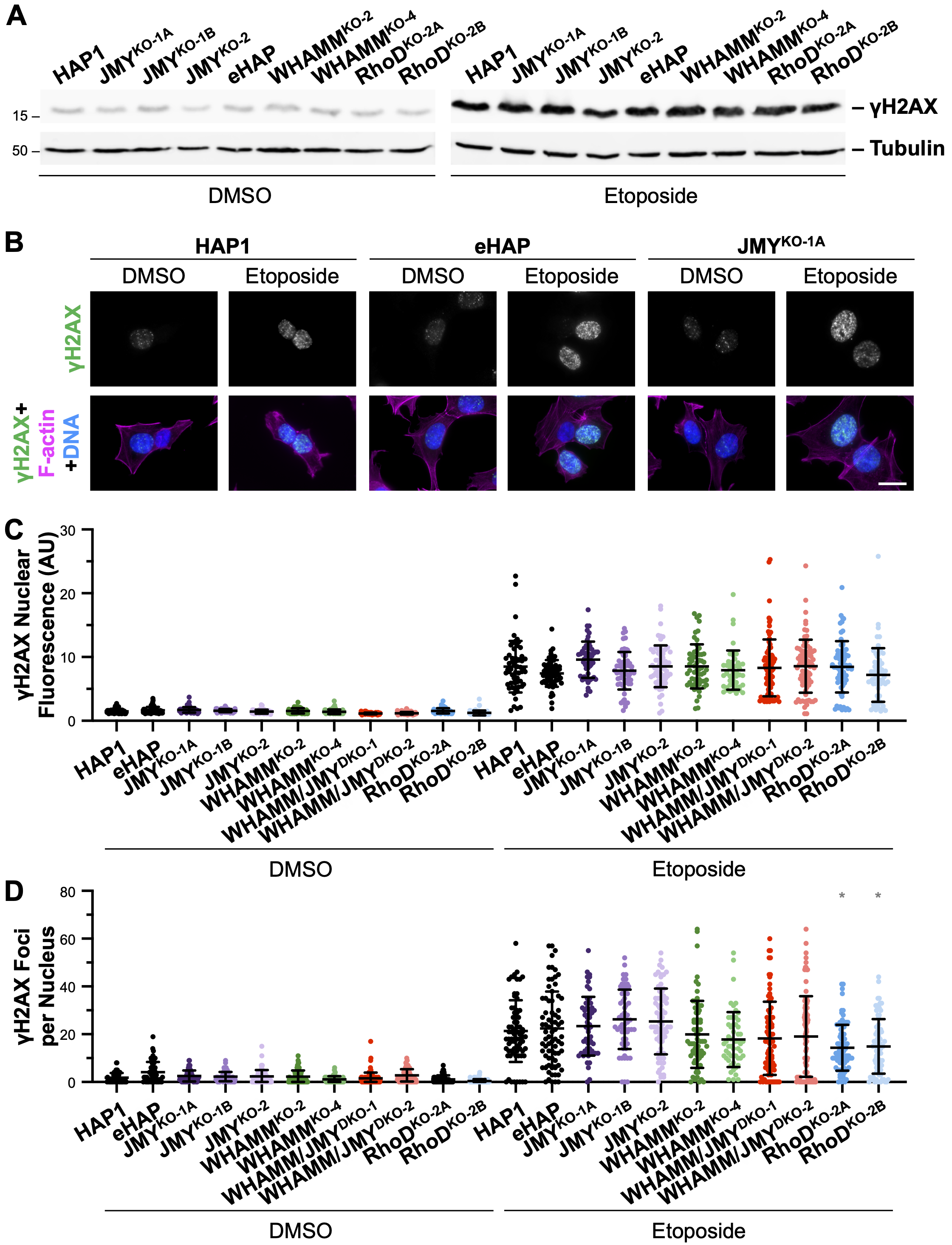

Supplement: S4 Fig — (A) HAP1, eHAP, and knockout cell lines were treated with DMSO or 5μM etoposide for 6h before immunoblotting with anti-γH2AX and anti-tubulin antibodies. (B) HAP1, eHAP, and JMYKO cells were treated with DMSO or etoposide, fixed, and stained with a γH2AX antibody (green), phalloidin (F-actin; magenta), and DAPI (DNA; blue). Representative images show increased nuclear γH2AX foci upon etoposide treatment. Scale bar, 25μm. (C) Nuclear γH2AX fluorescence intensity was calculated using ImageJ (n = 48–70 nuclei per sample from a representative experiment). (D) The number of γH2AX foci per nucleus was determined using ImageJ (n = 54–81 nuclei per sample from a representative experiment). Significance stars are in reference to the etoposide-treated eHAP cell line. Fewer γH2AX foci were observed in etoposide-treated RhoDKO samples because of the loss of some dead cells prior to fixation to the slide. *p<0.05 (ANOVA, Tukey post-hoc tests). (TIF) [file pgen.1009512.s007.tif]

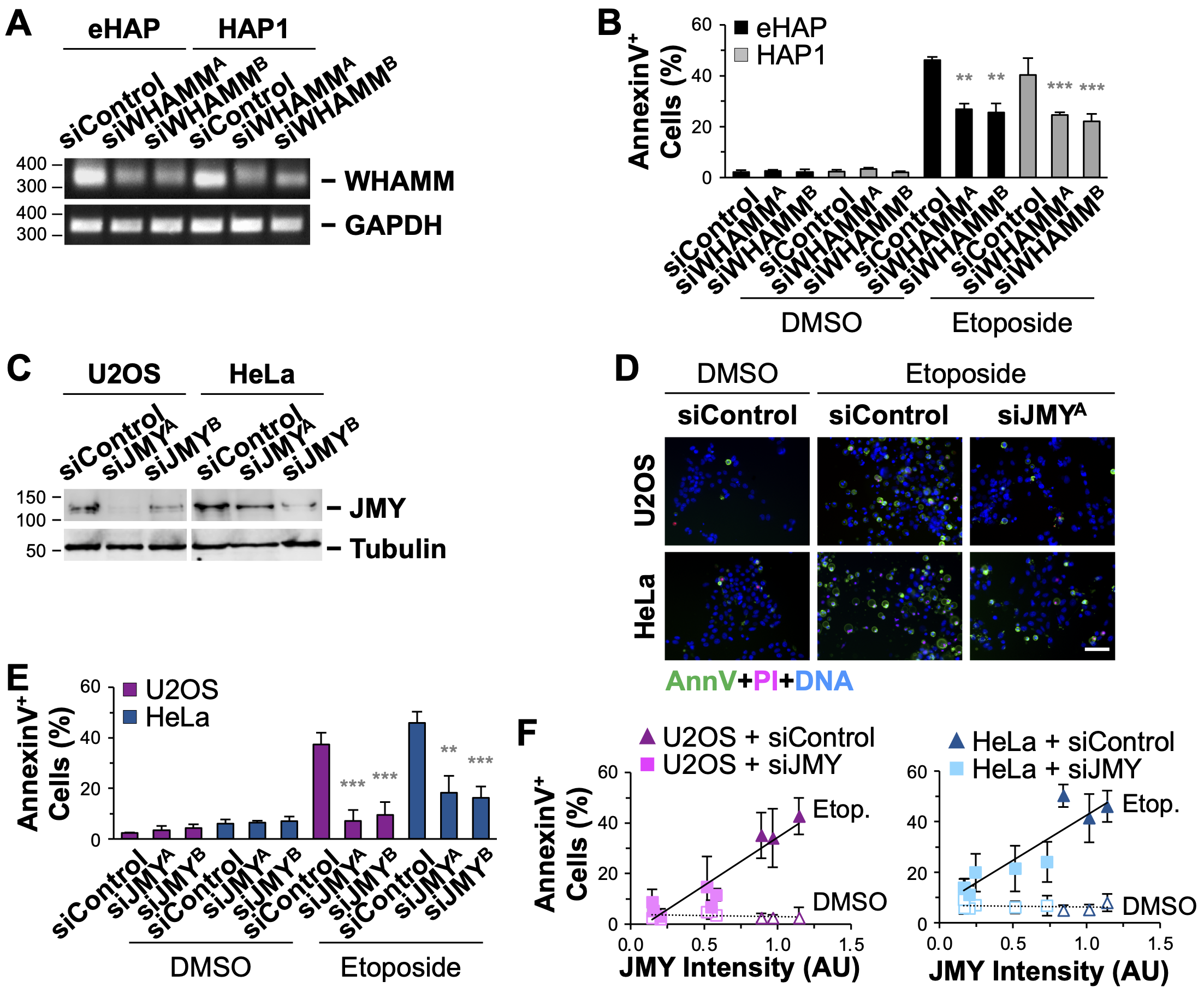

Supplement: S5 Fig — (A) eHAP or HAP1 cells were treated with control siRNAs or independent siRNAs for the WHAMM gene before collecting RNA and performing RT-PCR with primers for WHAMM and GAPDH. (B) The cells were treated with DMSO or 5μM etoposide for 6h and stained with Alexa488-AnnV, PI, and Hoechst. The % of AnnV-positive cells was calculated and each bar represents the mean ±SD from 3 experiments (n = 1,998–4,697 cells per bar). Significance stars refer to comparisons to the siControl samples. (C) U2OS or HeLa cells were treated with control siRNAs or independent siRNAs for the JMY gene before immunoblotting with antibodies to JMY and tubulin. (D) The cells were treated with DMSO or 10μM etoposide for 6h and stained with Alexa488-AnnV (green), PI (magenta), and Hoechst (blue). Scale bar, 100μm. (E) The % of AnnV-positive cells was calculated as in panel (B) (n = 476–866 cells per bar). (F) JMY band intensities on immunoblots were normalized to tubulin bands and plotted against the % of AnnV-positive cells. Each point represents the mean ±SD from 3 images in a given experiment (n = 69–440 cells per point). The slopes in the linear trendline regression equations for etoposide-treated samples (U2OS: Y = 38.31X - 3.86; HeLa: Y = 35.16X + 7.13) were significantly non-zero (p<0.001, R2>0.74). AU = Arbitrary Units. **p<0.01; ***p<0.001 (ANOVA, Tukey post-hoc tests). (TIF) [file pgen.1009512.s008.tif]

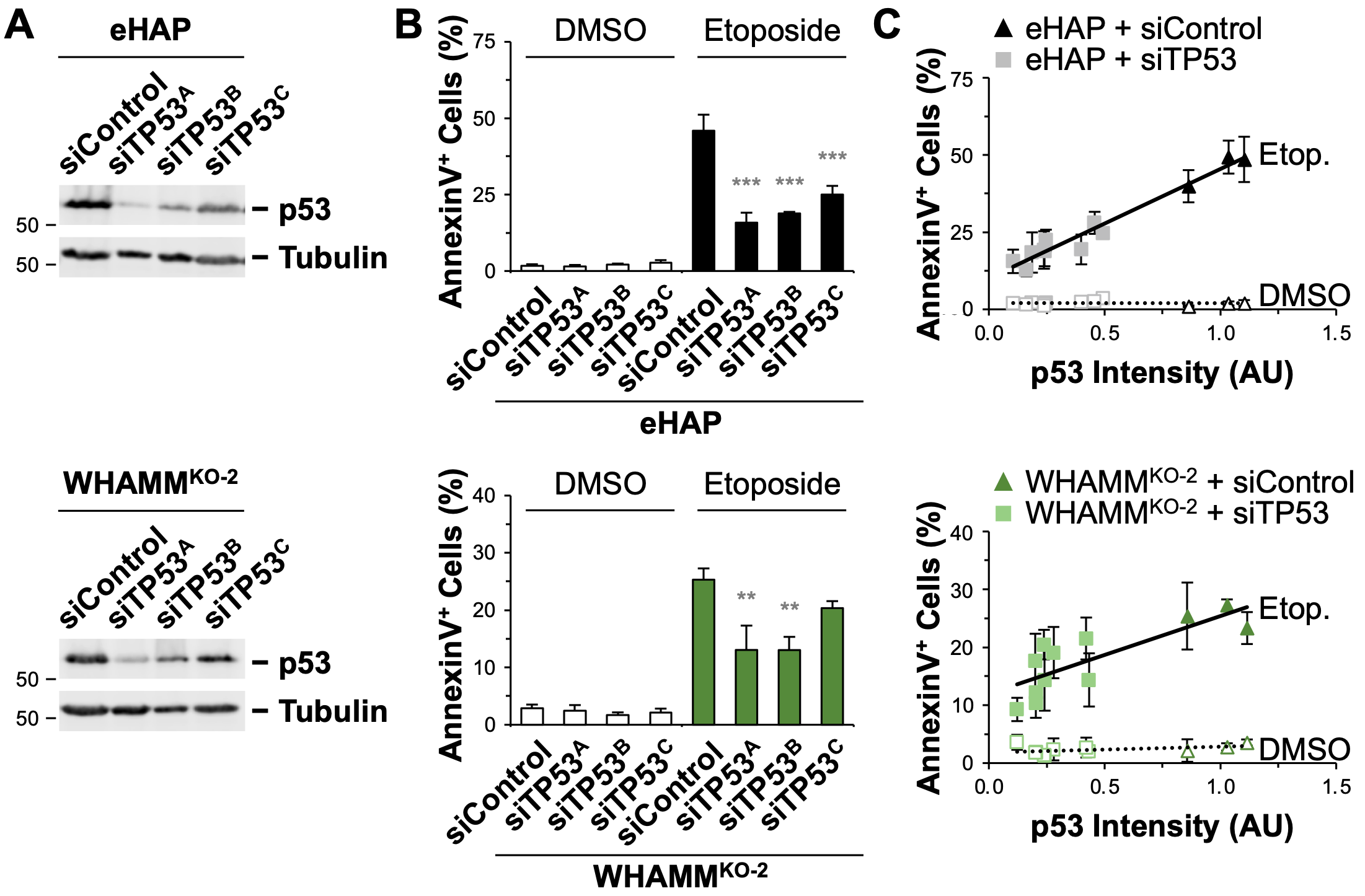

Supplement: S6 Fig — (A) eHAP and WHAMMKO-2 cells were treated with control siRNAs or independent siRNAs for the TP53 gene before immunoblotting with antibodies to p53 and tubulin. (B) The cells were treated with DMSO or 5μM etoposide for 6h and stained with Alexa488-AnnV, PI, and Hoechst. The % of AnnV-positive cells was calculated and each bar represents the mean ±SD from 3 experiments (n = 2,212–3,846 cells per bar). (C) p53 band intensities on immunoblots were normalized to tubulin bands and plotted against the % of AnnV-positive cells. Each point represents the mean ±SD from 3 fields-of-view in a given experiment (n = 576–1,509 cells per point). The slopes in the linear trendline regression equations for etoposide-treated samples (eHAP: Y = 35.44X + 10.99; WHAMMKO: Y = 13.28X + 11.81) were significantly non-zero (p<0.001, eHAP: R2 = 0.87; WHAMMKO: R2 = 0.47). AU = Arbitrary Units. These studies accompanied the experiments in Fig 5. **p<0.01; ***p<0.001 (ANOVA, Tukey post-hoc tests). (TIF) [file pgen.1009512.s009.tif]

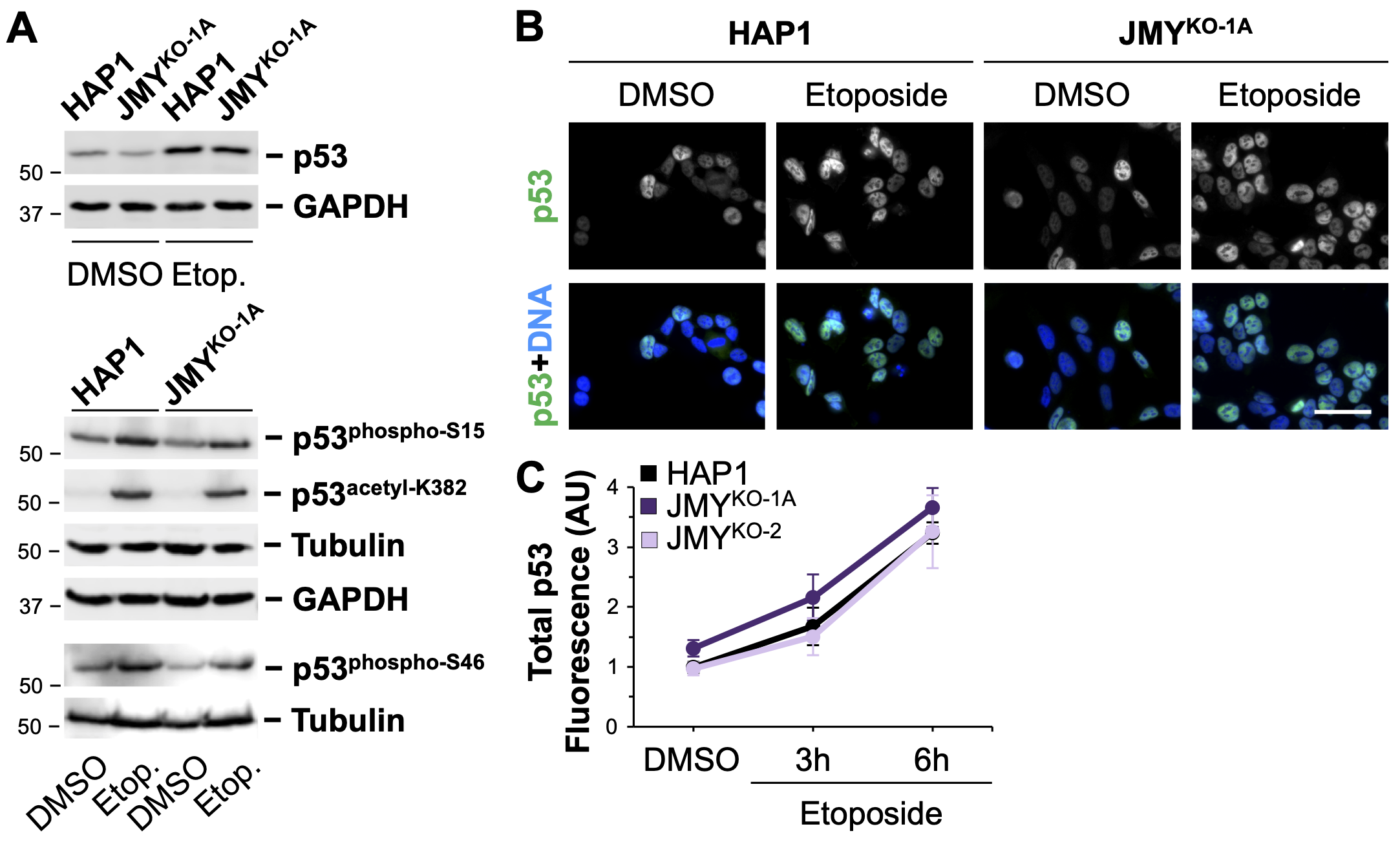

Supplement: S7 Fig — (A) HAP1 and JMYKO cells were treated with DMSO or 5μM etoposide for 6h before immunoblotting with antibodies to p53, p53phospho-S15, p53acetyl-K382, p53phospho-S46, GAPDH, and tubulin. (B) HAP1 and JMYKO cells were treated with DMSO or etoposide for 6h before being fixed and stained with a p53 antibody (green) and DAPI (DNA; blue). Scale bar, 30μm. (C) Cellular p53 fluorescence intensities were measured in ImageJ and the total p53 intensity was normalized to the HAP1 DMSO sample. Each bar represents the mean ±SD from 3 experiments (n = 196–343 cells per timepoint). AU = Arbitrary Units. (TIF) [file pgen.1009512.s010.tif]

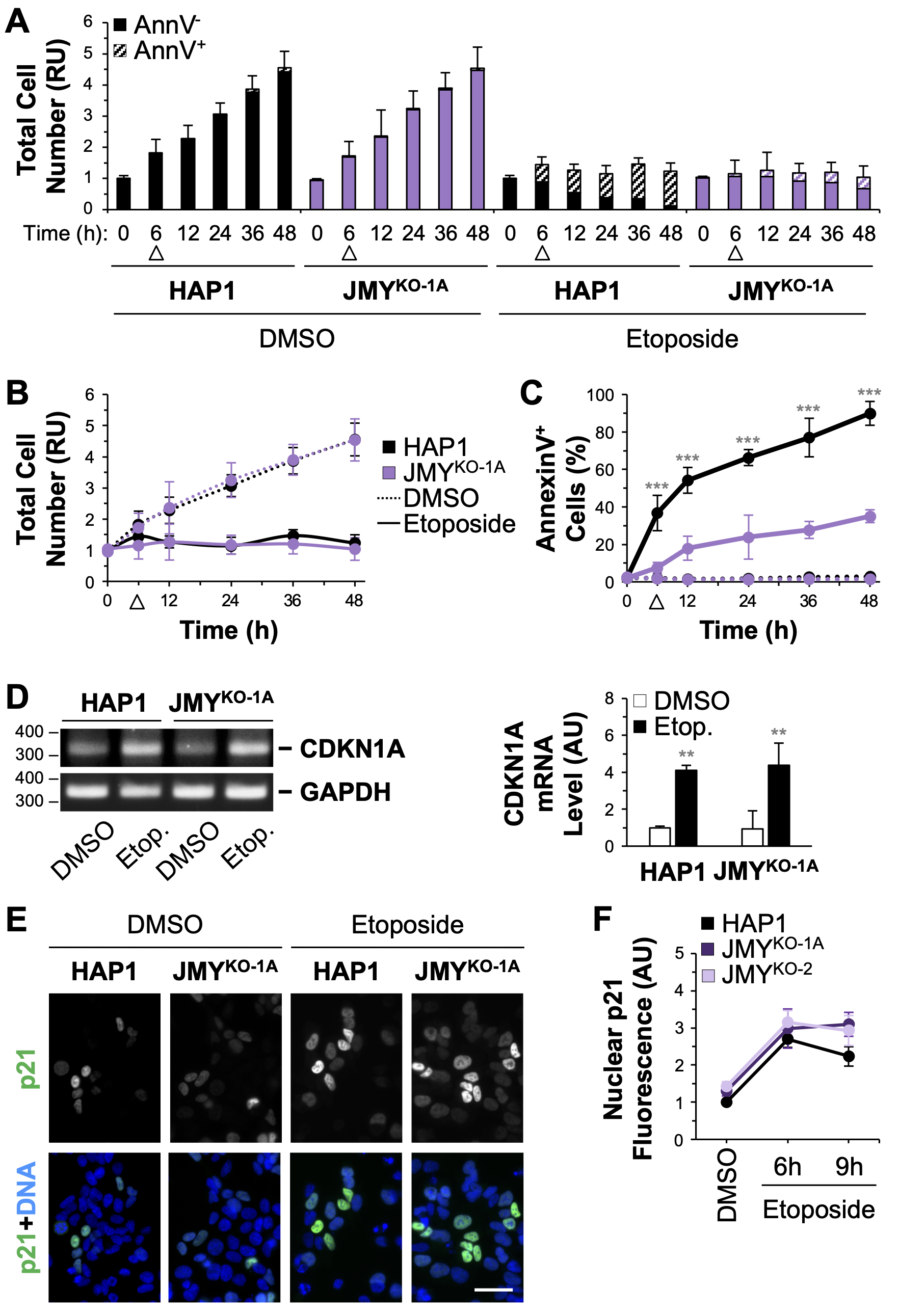

Supplement: S8 Fig — (A-C) HAP1 and JMYKO cells were treated with DMSO or 5μM etoposide for 6h before washout (triangles) and replacement with regular media. Samples were stained with Alexa488-AnnV, PI, and Hoechst at the indicated time points. In (A-B), the total # of cells (live and dead) was counted in ImageJ and normalized to the # at 0h for each sample. The total # of cells is displayed as the fraction of live AnnV-negative (AnnV-) or apoptotic AnnV-positive (AnnV+) cells (n = 1,461–9,108 cells per bar for DMSO; n = 1,415–2,992 cells per bar for etoposide). Panels (A) and (B) depict the same data in two different formats. In (C), the % of AnnV-positive cells was calculated and each point represents the mean ±SD from 3 experiments. Significance stars refer to comparisons among the etoposide-treated samples. RU = Relative Units. (D) HAP1 and JMYKO cells were treated with DMSO or etoposide for 6h before collecting RNA and performing RT-PCR with primers for CDKN1A and GAPDH. Agarose gel band intensities were quantified in ImageJ, and values for CDKN1A were normalized to GAPDH and plotted in the adjacent bar graph. AU = Arbitrary Units. (E) Cells were treated with DMSO or etoposide for 6h before being fixed and stained with a p21 antibody (green) and DAPI (DNA; blue). Scale bar, 30μm. (F) Nuclear p21 fluorescence intensity was measured in ImageJ, and each point represents the mean ±SD from 3 fields-of-view in a representative experiment (n = 216–418 cells per point). **p<0.01; ***p<0.001 (ANOVA, Tukey post-hoc tests). (TIF) [file pgen.1009512.s011.tif]

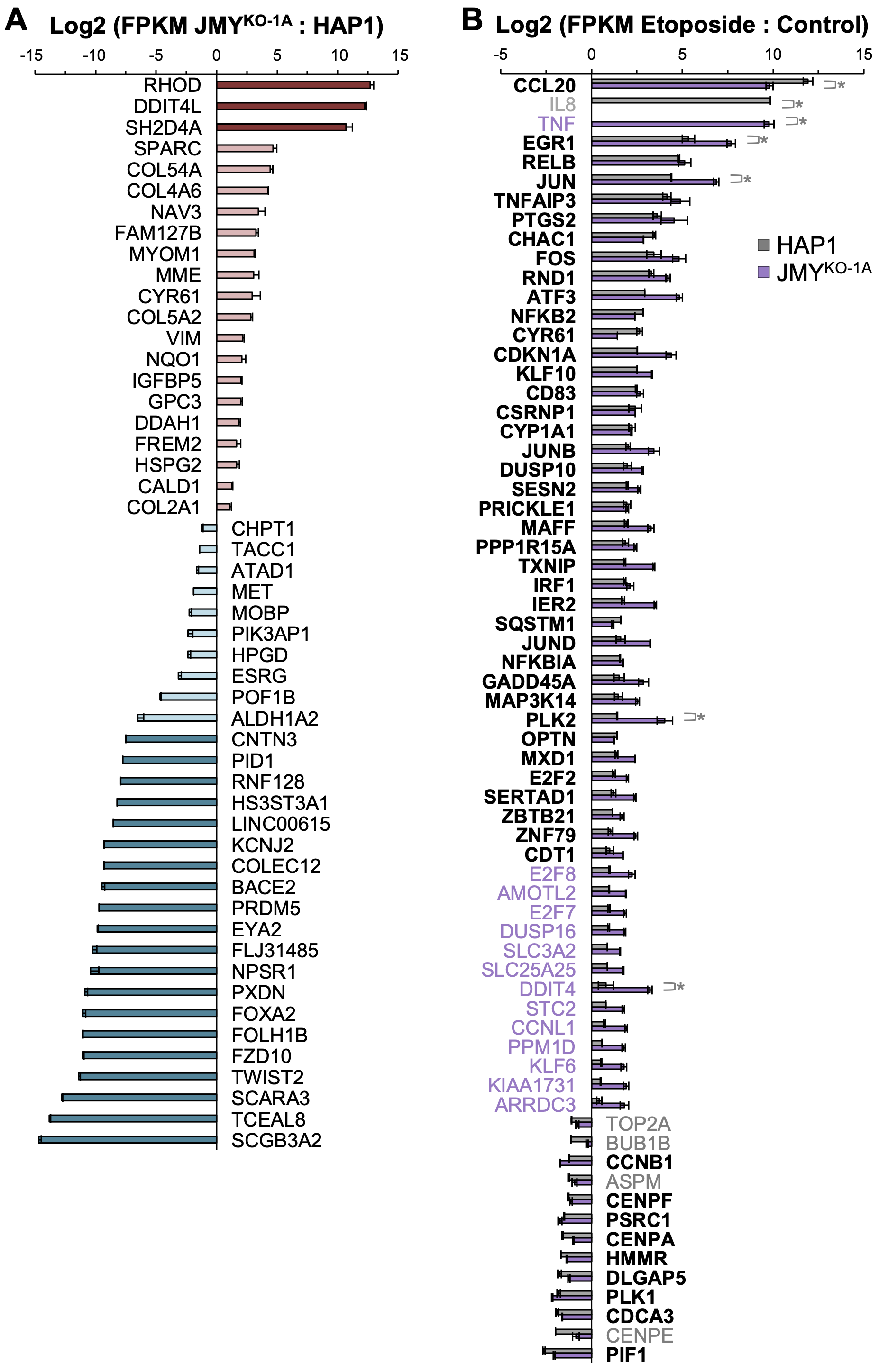

Supplement: S9 Fig — (A) RNA collected from HAP1 and JMYKO cells was subjected to mRNA sequencing analysis. FPKM values for individual genes with expression differences in JMYKO-1A vs HAP1 samples of at least 2-fold and with a significance q-value of <0.05 are shown in dark red (turned on), pink (up-regulated), light blue (down-regulated), or dark blue (turned off). Each bar represents the mean value ±SD from 3 independent RNA samples per genotype. (B) RNA collected from HAP1 and JMYKO cells treated with 5μM etoposide for 6h was subjected to mRNA sequencing analysis and comparisons to control samples from (A). FPKM values for individual genes with expression differences of at least 2-fold and with a significance q-value of <0.05 in etoposide vs control-treated HAP1 (gray bars) or JMYKO-1A (purple bars) samples are shown. Each bar represents the mean value ±SD from 2 independent RNA samples per genotype. Bolded black gene names indicate a >2-fold expression difference in both HAP1 and JMYKO cells, purple gene names indicate a >2-fold expression difference in JMYKO cells only, and gray gene names indicate a >2-fold expression difference in HAP1 cells only. Stars indicate differences of >2 in Log2(FPKM) values between HAP1 and JMYKO cells, with IL8 (in one replicate) and CCL20 (in both replicates) upregulated more in HAP1 cells, and TNF, EGR1, JUN, PLK2, and DDIT4 upregulated more in JMYKO samples. (TIF) [file pgen.1009512.s012.tif]

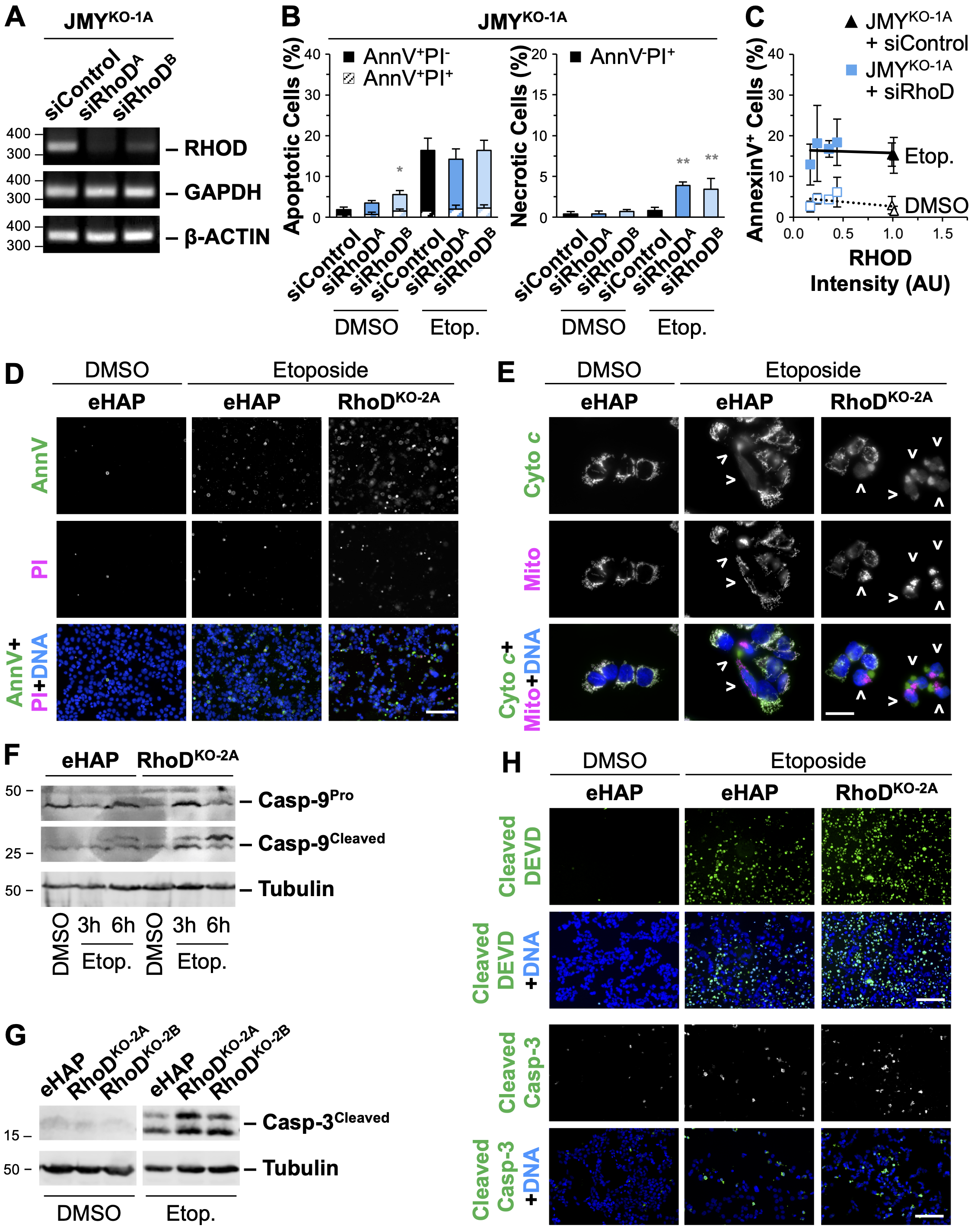

Supplement: S11 Fig — (A) JMYKO-1A cells were treated with control siRNAs or independent siRNAs for the RHOD gene before performing RT-PCRs with primers to RHOD, GAPDH, and β-ACTIN. (B) Cells were treated with DMSO or 5μM etoposide for 6h and stained with Alexa488-AnnV, PI, and Hoechst. The % of AnnV-positive cells was calculated and displayed as the fraction of AnnV-positive/PI-negative (AnnV+PI-) or AnnV/PI double-positive (AnnV+PI+) cells. Significance stars refer to comparisons of total AnnV+ counts for siControl vs siRhoD samples. The % of AnnV-negative/PI-positive (AnnV-PI+) necrotic cells was also quantified. Each bar represents the mean ±SD from 3 experiments (n = 3,166–4,998 cells per sample). (C) RHOD band intensities were normalized to β-ACTIN and plotted versus the % of AnnV-positive cells. Each point represents the mean ±SD from 3 fields-of-view in a given experiment (n = 670–1,919 cells per point). (D) eHAP and RhoDKO cells were treated with DMSO or etoposide and stained with Alexa488-AnnV (green), PI (magenta), and Hoechst (DNA; blue). Scale bar, 100μm. (E) Cells were treated with DMSO or etoposide, fixed, and stained with antibodies to detect cytochrome c (Cyto c; green), AIF (Mito; magenta), and DAPI (DNA; blue). Scale bar, 25μm. (F-G) Cells were treated with DMSO for 6h or etoposide for 3 or 6h, and extracts were immunoblotted with antibodies to caspase-9 (Casp-9Pro and Casp-9Cleaved), caspase-3 (Casp-3Cleaved), and tubulin. (H) eHAP and RhoDKO cells were treated with DMSO or etoposide for 6h and stained with caspase-3/7 green detection reagent (Cleaved DEVD; green) and Hoechst (DNA; blue), or fixed and stained with an antibody that recognizes active caspase-3 cleaved at Asp175 (Cleaved Casp-3; green) and DAPI (DNA; blue). Scale bars, 100μm. *p<0.05, **p<0.01 (ANOVA, Tukey post-hoc tests). (TIF) [file pgen.1009512.s014.tif]

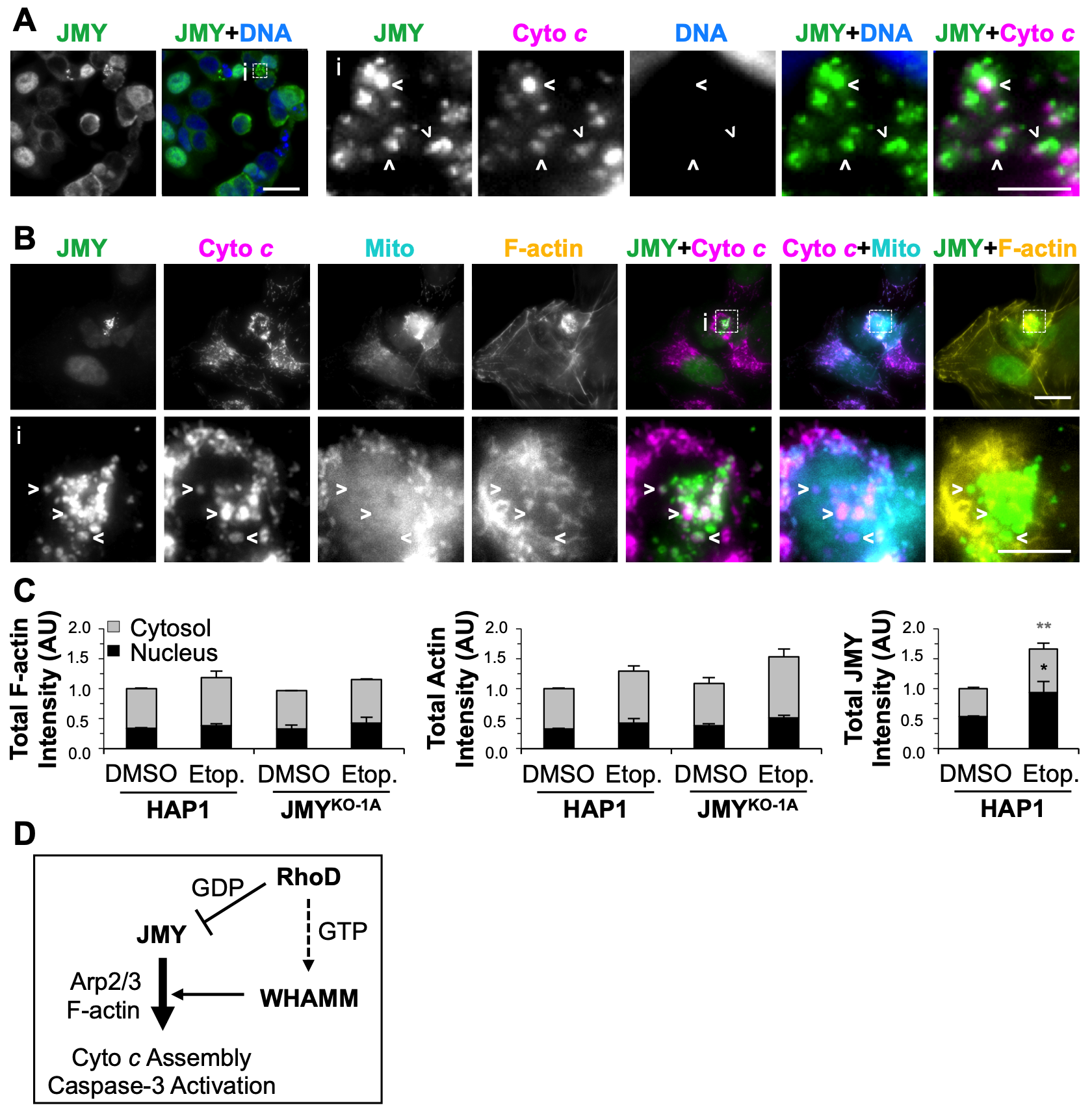

Supplement: S12 Fig — (A) HAP1 cells were treated with 5μM etoposide for 6h before being fixed and stained with a JMY antibody (green), a cyto c antibody (magenta), and DAPI (DNA; blue). Arrowheads and magnifications represent examples of juxtanuclear JMY and cyto c puncta. Scale bars, 25μm, 10μm. (B) U2OS cells were treated with 10μM etoposide for 6h before being incubated with MitoTracker (Mito; cyan), fixed, and stained with a JMY antibody (green), a cyto c antibody (magenta), and phalloidin (F-actin; yellow). Arrowheads and magnifications depict clusters of JMY and cyto c puncta in an F-actin territory. Scale bars, 25μm, 10μm. (C) HAP1 and JMYKO cells were treated with 5μM etoposide for 6h before being fixed and stained with a JMY antibody, DAPI, and either phalloidin to visualize F-actin or an actin antibody to visualize total actin. Nuclear and cytoplasmic F-actin, total actin, and JMY fluorescence intensities were measured in ImageJ and the total intensity was normalized to the HAP1 DMSO sample. Each bar represents the mean ±SD from 2–4 experiments (F-actin: n = 106–163 cells per bar; Total actin: n = 103–148 cells per bar; JMY: n = 234–311 cells per bar). Gray significance stars refer to comparisons of the cytoplasmic intensity to the DMSO samples and black significance stars refer to comparisons of the nuclear intensity to the DMSO samples. AU = Arbitrary Units. *p<0.05, **p<0.01 (t-test). (D) Model for JMY, WHAMM, the Arp2/3 complex, F-actin, and RhoD in apoptosis. Line thickness reflects different degrees of apoptosis-related activities, line dashing represents a potential role for RhoD based on interactions shown in the literature. (TIF) [file pgen.1009512.s015.tif]
