# Supplementary material for: The actin nucleation factors JMY and WHAMM enable a rapid Arp2/3 complex-mediated intrinsic pathway of apoptosis
Source: PLoS Genet. 2021 Apr 19;17(4):e1009512. doi: 10.1371/journal.pgen.1009512 (PMC8084344; doi:10.1371/journal.pgen.1009512)
Supplement: S10 Fig — RNA collected from HAP1 and JMYKO cells was subjected to mRNA sequencing analysis as in Fig 6. Data for several classes of gene products involved in apoptosis or cytoskeletal regulation are shown. For etoposide-treated samples, genes related to TNF or other signaling pathways are also included. (PDF) [file pgen.1009512.s013.pdf]

Gene		Log2 (FPKM JMY^{KO-1A} : HAP1)		q-value
		Mean	StDev	
JMY Interactors	EP300	0.17	0.02	0.999
	STRAP	-0.31	0.13	0.999
	TP53	0.57	0.08	0.999
	MDM2	-0.04	0.05	0.999
Apoptotic Caspases	CASP2	0.09	0.14	0.999
	CASP3	-0.16	0.12	0.999
	CASP6	-0.31	0.01	0.999
	CASP7	-0.19	0.05	0.999
	CASP8	-0.04	0.14	0.999
	CASP9	-0.26	0.13	0.999
Apoptosis Regulators	BIRC2	-0.27	0.20	0.999
	BIRC5	0.05	0.07	0.999
	BIRC6	0.12	0.07	0.999
	BCL2	-0.39	0.33	0.999
	BCL2L1	0.50	0.04	0.999
	BCL2L10	-0.04	0.25	0.999
	BCL2L12	0.26	0.15	0.999
	MCL1	-0.33	0.04	0.999
	BAX	-0.11	0.08	0.999
	BAK1	-0.06	0.13	0.999
	BOK	0.24	0.16	0.999
	BID	-0.05	0.04	0.999
	BCL2L11	-0.20	0.17	0.999
	BMF	0.85	0.03	0.999
	BAD	0.00	0.48	0.999
	BIK	-0.95	0.27	0.999
	PMAIP1	0.09	0.14	0.999
	BBC3	0.77	0.28	0.999
	APAF1	-0.14	0.04	0.999
	CYCS	-0.17	0.26	0.999
Cell Cycle Inhibitors	CDKN1A	0.10	0.65	0.999
	CDKN2A	0.10	0.35	0.999
Nucleation Promoting Factors	WASL	-0.50	0.08	0.999
	WASH1	0.20	0.07	0.999
	WASF1	-0.43	0.06	0.999
	WASF2	0.06	0.06	0.999
	WASF3	-0.13	0.20	0.999
	WHAMM	-0.08	0.01	0.999
	JMY	0.05	0.29	0.999
	NCKIPSD	-0.09	0.17	0.999
	CTTN	0.06	0.06	0.999
Tandem Actin Monomer-binding Proteins of Nucleation	COBL	-0.03	0.08	0.999
	SPIRE1	0.08	0.09	0.999
	SPIRE2	0.77	0.35	0.999
Formins	APC	-0.11	0.06	0.999
	DIAPH1	-0.18	0.02	0.999
	DIAPH2	0.05	0.06	0.999
	DIAPH3	-0.07	0.13	0.999
	DAAM1	-0.29	0.14	0.999
	FMN2	0.67	0.12	0.999
	FMNL2	-0.44	0.12	0.999
	FMNL3	0.27	0.06	0.999
	FHOD1	0.06	0.22	0.999
	FHOD3	0.76	0.16	0.999
	INF2	0.19	0.36	0.999
Arp2/3 Complex	ACTR2	-0.05	0.21	0.999
	ACTR3	0.04	0.16	0.999
	ARPC1A	-0.01	0.12	0.999
	ARPC1B	-0.02	0.33	0.999
	ARPC2	-0.24	0.06	0.999
	ARPC3	-0.22	0.21	0.999
	ARPC4	-0.11	0.03	0.999
	ARCP5	-0.23	0.03	0.999
	ARCP5L	-0.13	0.12	0.999

Gene		Log2(FPKM JMY^{KO-1A} : HAP1)		q-value
		Mean	StDev	
Ras	DIRAS1	0.21	0.25	0.999
	GEM	-0.31	0.55	0.999
	HRAS	0.13	0.12	0.999
	KRAS	-0.08	0.10	0.999
	MRAS	0.69	0.09	0.999
	NRAS	-0.21	0.12	0.999
	NKIRAS1	0.00	0.24	0.999
	NKIRAS2	0.07	0.13	0.999
	RRAS	0.35	0.10	0.999
	RALA	-0.22	0.18	0.999
	RALB	0.09	0.16	0.999
	RAP1A	-0.22	0.25	0.999
	RAP1B	-0.08	0.21	0.999
	RAP2A	-0.19	0.14	0.999
	RAP2B	0.06	0.23	0.999
	RAP2C	-0.04	0.22	0.999
	RASLL10B	0.85	0.05	0.999
	RASL11A	-0.41	0.18	0.999
	RASL11B	-0.78	0.19	0.999
	RHEB	-0.13	0.12	0.999
	RHEBL1	-0.14	0.36	0.999
	RIT1	-0.24	0.14	0.999
	RRAS2	0.07	0.20	0.999
Arf	ARF1	0.08	0.03	0.999
	ARF3	0.20	0.09	0.999
	ARF4	0.08	0.14	0.999
	ARL4D	0.24	0.37	0.999
	ARF5	0.07	0.09	0.999
	ARF6	-0.14	0.05	0.999
	ARFRP1	0.10	0.22	0.999
	ARFRP2	-0.74	0.20	0.999
	ARL16	0.23	0.08	0.999
	ARL2	-0.27	0.12	0.999
	ARL2BP	-0.03	0.11	0.999
	ARL3	0.23	0.18	0.999
	ARL4A	-0.15	0.14	0.999
	ARL5A	-0.04	0.32	0.999
	ARL6	-0.22	0.07	0.999
	ARL4C	0.09	0.09	0.999
	ARL8A	-0.21	0.21	0.999
	SAR1A	-0.12	0.12	0.999
	SAR1B	-0.05	0.18	0.999
Rho	CDC42	-0.01	0.14	0.999
	RAC1	-0.08	0.06	0.999
	RAC2	1.67	0.77	0.999
	RAC3	-0.02	0.08	0.999
	RHOA	-0.01	0.09	0.999
	RHOB	-1.82	0.76	0.999
	RHOBTB1	-0.13	0.08	0.999
	RHOBTB2	-0.63	0.08	0.999
	RHOBTB3	-0.20	0.17	0.999
	RHOC	0.50	0.12	0.999
	RHOD	12.70	0.28	0.013
	RHOF	0.26	0.18	0.999
	RHOG	0.01	0.22	0.999
	RHOQ	-0.17	0.15	0.999
	RHOU	-0.62	0.20	0.999
	RHOV	-0.12	0.13	0.999
	RIF1	-0.06	0.18	0.999
	RND1	0.02	0.07	0.999
	RND2	0.19	0.14	0.999
	RAN	-0.10	0.08	0.999

Gene		Log2 (FPKM JMY^{KO-1A} : HAP1)		q-value
		Mean	StDev	
Rab	RAB1A	0.05	0.12	0.999
	RAB1B	-0.10	0.09	0.999
	RAB2A	-0.34	0.11	0.999
	RAB2B	0.02	0.04	0.999
	RAB3A	-0.25	0.42	0.999
	RAB3B	-0.40	0.04	0.999
	RAB3D	-0.20	0.13	0.999
	RAB4A	-0.17	0.12	0.999
	RAB4B	-0.49	0.47	0.999
	RAB5A	-0.05	0.04	0.999
	RAB5B	-0.12	0.03	0.999
	RAB5C	0.01	0.06	0.999
	RAB6A	-0.11	0.02	0.999
	RAB6B	-0.06	0.08	0.999
	RAB7A	-0.30	0.08	0.999
	RAB7L1	-1.00	0.11	0.589
	RAB8A	-0.07	0.09	0.999
	RAB8B	0.38	0.22	0.999
	RAB9A	-0.39	0.22	0.999
	RAB9B	-1.02	0.31	0.999
	RAB10	-0.17	0.12	0.999
	RAB11A	-0.11	0.16	0.999
	RAB11B	0.18	0.29	0.999
	RAB12	-0.16	0.17	0.999
	RAB13	0.06	0.18	0.999
	RAB14	-0.15	0.10	0.999
	RAB15	-0.88	0.14	0.648
	RAB18	0.00	0.19	0.999
	RAB20	-0.59	0.34	0.999
	RAB21	0.15	0.19	0.999
	RAB22A	-0.48	0.10	0.999
	RAB23	-0.35	0.13	0.999
	RAB24	0.05	0.19	0.999
	RAB27A	-0.11	0.25	0.999
	RAB28	-0.07	0.16	0.999
	RAB30	0.27	0.14	0.999
	RAB31	-0.06	0.05	0.999
	RAB33A	-0.76	0.41	0.999
	RAB33B	-0.02	0.06	0.999
	RAB34	1.28	0.16	0.999
	RAB35	-0.04	0.08	0.999
	RAB38	-0.62	0.04	0.999
	RAB39A	-0.18	0.79	0.999
	RAB40A	0.84	0.61	0.999
	RAB40B	-0.25	0.07	0.999
	RAB40C	0.19	0.26	0.999
	RAB42	0.17	0.22	0.999

	Log2 (FPKM)	q-value
Unchanged	-----	>0.05
Turned On	>10	<0.05
Up-regulated	>1	<0.05
Down-regulated	<-1	<0.05
Turned Off	<-10	<0.05

Gene		HAP1 Etop. : Control			JMY^{KO-1A} Etop. : Control		
		Log2 (FPKM)		q-value	Log2 (FPKM)		q-value
		Mean	StDev		Mean	StDev	
JMY Interactors	EP300	-0.01	0.06	0.999	0.59	0.06	0.999
	STRAP	-0.28	0.05	0.999	-0.26	0.11	0.999
	TP53	0.69	0.10	0.999	0.51	0.08	0.999
	MDM2	0.13	0.01	0.999	1.05	0.03	0.691
Apoptotic Caspases	CASP2	0.42	0.01	0.999	0.75	0.08	0.999
	CASP3	0.06	0.13	0.999	0.19	0.11	0.999
	CASP6	-0.18	0.06	0.999	-0.51	0.01	0.999
	CASP7	0.18	0.03	0.999	0.17	0.01	0.999
	CASP8	0.24	0.34	0.999	0.07	0.10	0.999
	CASP9	0.35	0.27	0.999	0.50	0.42	0.999
Apoptosis Regulators	BIRC2	0.32	0.01	0.999	0.51	0.06	0.999
	BIRC5	-0.63	0.03	0.999	-0.70	0.08	0.999
	BIRC6	-0.48	0.03	0.999	-0.37	0.00	0.999
	BCL2	0.27	0.52	0.999	0.16	0.57	0.999
	BCL2L1	-0.07	0.09	0.999	-0.28	0.09	0.999
	BCL2L10	0.55	0.35	0.999	0.92	0.13	0.999
	BCL2L12	0.13	0.04	0.999	0.25	0.14	0.999
	MCL1	0.13	0.02	0.999	0.84	0.09	0.932
	BAX	-0.06	0.02	0.999	-0.07	0.05	0.999
	BAK1	0.09	0.10	0.999	0.02	0.01	0.999
	BOK	1.12	0.11	0.999	0.72	0.05	0.999
	BID	0.01	0.02	0.999	-0.28	0.04	0.999
	BCL2L11	0.20	0.12	0.999	0.00	0.04	0.999
	BMF	0.86	0.14	0.999	0.56	0.03	0.999
	BAD	0.14	0.23	0.999	0.25	0.03	0.999
	BIK	0.84	0.18	0.999	1.03	0.04	0.999
	PMAIP1	1.20	0.04	0.325	1.85	0.21	0.089
	BBC3	1.32	0.46	0.748	2.26	0.10	0.114
	APAF1	-0.22	0.08	0.999	-0.01	0.13	0.999
	CYCS	-0.10	0.10	0.999	-0.08	0.13	0.999
Cell Cycle Inhibitors	CDKN1A	2.53	0.01	0.016	4.39	0.28	0.016
	CDKN2A	0.14	0.07	0.999	0.05	0.26	0.999
Nucleation Promoting Factors	WASL	-0.32	0.01	0.999	0.32	0.16	0.999
	WASH1	0.80	0.45	0.999	0.79	0.63	0.999
	WASF1	-0.30	0.01	0.999	0.06	0.02	0.999
	WASF2	0.03	0.02	0.999	0.06	0.06	0.999
	WASF3	-0.36	0.11	0.999	-0.43	0.16	0.999
	WHAMM	0.82	0.11	0.900	1.73	0.06	0.075
	JMY	0.21	0.03	0.999	0.23	0.05	0.999
	NCKIPSD	0.38	0.08	0.999	0.52	0.06	0.999
	CTTN	-0.11	0.05	0.999	-0.28	0.01	0.999
Tandem Actin Monomer-binding Proteins of Nucleation	COBL	-0.23	0.06	0.999	-0.12	0.05	0.999
	SPIRE1	-0.22	0.04	0.999	-0.33	0.03	0.999
	SPIRE2	0.55	0.10	0.999	1.06	0.29	0.995
Formins	APC	-0.28	0.13	0.999	-0.13	0.03	0.999
	DIAPH1	0.02	0.09	0.999	0.05	0.01	0.999
	DIAPH2	-0.87	0.13	0.771	-0.64	0.07	0.999
	DIAPH3	-0.60	0.08	0.999	-0.02	0.04	0.999
	DAAM1	-0.01	0.18	0.999	0.33	0.04	0.999
	FMN2	-0.86	0.11	0.836	0.61	0.04	0.999
	FMNL2				-1.03	0.04	0.923
	FMNL3	0.11	0.03	0.999	0.07	0.01	0.999
	FHOD1	-0.12	0.04	0.999	-0.12	0.04	0.999
	FHOD3	-0.24	0.04	0.999	-0.24	0.04	0.999
	INF2	0.18	0.27	0.999	0.21	0.04	0.999
Arp2/3 Complex	ACTR2	-0.23	0.05	0.999	-0.26	0.15	0.999
	ACTR3	-0.32	0.02	0.999	-0.32	0.13	0.999
	ARPC1A	-0.05	0.01	0.999	-0.37	0.04	0.999
	ARPC1B	0.11	0.05	0.999	-0.12	0.01	0.999
	ARPC2	-0.23	0.02	0.999	-0.36	0.08	0.999
	ARPC3	-0.21	0.05	0.999	-0.24	0.10	0.999
	ARPC4	-0.01	0.01	0.999	-0.05	0.08	0.999
	ARCP5	-0.28	0.08	0.999	-0.24	0.13	0.999
	ARCP5L	0.02	0.11	0.999	-0.07	0.01	0.999
TNF Family and Signaling Molecules	ATF1	-0.07	0.01	0.999	0.48	0.04	0.999
	ATF2	-0.13	0.11	0.999	0.54	0.10	0.999
	ATF3	2.93	0.00	0.016	4.84	0.17	0.016
	ATF4	-0.09	0.06	0.999	-0.01	0.05	0.999
	ATF5	0.29	0.04	0.999	0.07	0.00	0.999
	ATF6	-0.11	0.11	0.999	-0.19	0.08	0.999
	ATF7	-0.25	0.02	0.999	0.10	0.49	0.999
	BAG4	-0.13	0.02	0.999	0.11	0.04	0.999
	CAV1	2.30	0.20	0.180	0.80	0.23	0.999
	CFLAR	0.06	0.01	0.999	0.05	0.04	0.999
	CHUK	-0.22	0.04	0.999	0.10	0.08	0.999
	CRADD	-0.35	0.13	0.999	-0.37	0.23	0.999
	EDARADD	0.01	0.01	0.999	-0.27	0.09	0.999
	ELK1	-0.04	0.13	0.999	-0.02	0.11	0.999
	FADD	-0.06	0.12	0.999	-0.03	0.09	0.999
	FAS	1.23	0.32	0.999	1.38	0.09	0.999
	FOS	3.44	0.40	0.219	4.83	0.36	0.016
	IKBKB	0.25	0.04	0.999	0.46	0.01	0.999
	IKBKG	0.22	0.13	0.999	0.57	0.05	0.999
	JUN	4.40	0.02	0.016	6.87	0.15	0.016
	JUNB	2.02	0.12	0.016	3.44	0.30	0.016
	JUND	1.61	0.25	0.023	3.24	0.01	0.016
	LTBR	0.45	0.10	0.999			
TNF Family and Signaling Molecules (cont.)	MADD	0.30	0.04	0.999	0.51	0.02	0.999
	MAP2K3	-0.26	0.02	0.999	-0.41	0.11	0.999
	MAP2K4	-0.09	0.04	0.999	-0.05	0.08	0.999
	MAP2K7	-0.03	0.28	0.999	0.19	0.03	0.999
	MAP3K1	-0.03	0.09	0.999	0.53	0.08	0.999
	MAP3K14	1.49	0.22	0.434	2.54	0.11	0.016
	MAP3K2	0.03	0.04	0.999	0.30	0.04	0.999
	MAP3K3	0.05	0.00	0.999	0.27	0.06	0.999
	MAP3K4	-0.42	0.06	0.999	0.21	0.01	0.999
	MAP3K5	-0.66	0.04	0.999	-0.50	0.04	0.999
	MAP3K7	0.81	0.29	0.999	0.50	0.05	0.999
	MAP4K2	-0.04	0.01	0.999	0.09	0.04	0.999
	MAP4K3	-0.64	0.01	0.999	-0.32	0.06	0.999
	MAP4K4	-0.24	0.02	0.999	-0.38	0.01	0.999
	MAP4K5	-0.41	0.06	0.999	0.07	0.10	0.999
	MAPK1	-0.22	0.07	0.999	-0.08	0.01	0.999
	MAPK11	0.07	0.33	0.999	-0.36	0.33	0.999
	MAPK12	0.11	0.17	0.999	-0.24	0.03	0.999
	MAPK14	-0.17	0.02	0.999	-0.09	0.06	0.999
	MAPK3	0.02	0.06	0.999	-0.34	0.05	0.999
	MAPK4	0.80	0.23	0.999	1.11	0.53	0.968
	MAPK6	-0.05	0.04	0.999	-0.05	0.03	0.999
	MAPK7	0.49	0.12	0.999	0.60	0.19	0.999
	MAPK8	-0.14	0.13	0.999	0.14	0.14	0.999
	NFKB1	0.78	0.02	0.910	0.82	0.10	0.987
	NFKB2	2.84	0.01	0.016	2.39	0.01	0.016
	NFKBIA	1.57	0.03	0.016	1.73	0.02	0.046
	NFKBIB	0.49	0.03	0.999	0.47	0.10	0.999
	NFKBID	0.57	0.22	0.999	-0.08	0.64	0.999
	NKFBIE	0.89	0.09	0.999	1.45	0.21	0.608
	NGFR	0.72	0.11	0.999	0.19	0.10	0.999
	NSMAF	-0.25	0.06	0.999	-0.17	0.16	0.999
	OTUD7B	0.09	0.06	0.999	0.37	0.03	0.999
	PRKCI	-0.19	0.07	0.999	-0.16	0.11	0.999
	PRKCZ	0.16	0.18	0.999	0.05	0.07	0.999
	RBCK1	0.17	0.14	0.999	0.35	0.04	0.999
	RELA	0.32	0.08	0.999	0.44	0.04	0.999
	RELB	4.81	0.05	0.016	5.13	0.35	0.016
	RELT	0.53	0.06	0.999	0.60	0.07	0.999
	RFFL	-0.07	0.13	0.999	0.31	0.01	0.999
	RIPK1	0.54	0.01	0.999	0.85	0.08	0.999
	RNF31	0.15	0.10	0.999	-0.02	0.08	0.999
	RPS27A	-0.09	0.15	0.999	-0.30	0.05	0.999
	SHARPIN	0.14	0.10	0.999	-0.01	0.13	0.999
	SMPD1	0.70	0.02	0.999	0.17	0.05	0.999
	SMPD2	0.08	0.13	0.999	-0.21	0.44	0.999
	SMPD4	0.16	0.12	0.999	0.22	0.04	0.999
	SPPL2A	0.05	0.01	0.999	0.22	0.11	0.999
	SPPL2B	0.48	0.23	0.999	0.41	0.14	0.999
	SQSTM1	1.63	0.01	0.016	1.17	0.06	0.793
	STAT1	0.06	0.09	0.999	0.03	0.06	0.999
	TAB1	-0.12	0.11	0.999	-0.21	0.04	0.999
	TAB2	-0.03	0.01	0.999	0.27	0.03	0.999
	TAB3	-0.05	0.13	0.999	0.10	0.00	0.999
	TANK	0.27	0.04	0.999	0.33	0.01	0.999
	TAX1BP1	-0.15	0.04	0.999	-0.13	0.01	0.999
	TNF				9.78	0.27	0.023
	TNFAIP3	4.15	0.23	0.016	4.89	0.52	0.016
	TNFRSF10A	-0.12	0.04	0.999	-0.15	0.01	0.999
	TNFRSF10B	0.35	0.03	0.999	0.75	0.03	0.999
	TNFRSF10D	0.15	0.19	0.999	-0.24	0.09	0.999
	TNFRSF11A	-0.01	0.05	0.999	-0.14	0.04	0.999
	TNFRSF12A	1.45	0.01	0.915	2.08	0.11	0.248
	TNFRSF19	0.35	0.25	0.999	-0.16	0.06	0.999
	TNFRSF1A	0.59	0.09	0.999	0.16	0.33	0.999
	TNFRSF1B	0.25	0.43	0.999			
	TNFRSF21	-0.24	0.01	0.999	-0.70	0.25	0.999
	TNFRSF5	0.19	0.05	0.999	-0.43	0.08	0.999
	TNFSF10	0.98	0.28	0.999			
	TNFSF12	0.11	0.63	0.999	-0.77	0.33	0.999
	TNFSF13	-0.73	0.12	0.999	-1.22	0.22	0.999
	TFNSF9	0.67	0.17	0.999	1.15	0.24	0.909
	TNIK	0.09	0.09	0.999	-0.33	0.05	0.999
	TRADD	-0.02	0.42	0.999	0.12	0.65	0.999
	TRAF2	-0.04	0.19	0.999	0.32	0.02	0.999
	TRAF3	0.12	0.24	0.999	0.19	0.03	0.999
	TRAF4	0.71	0.08	0.999	1.17	0.10	0.570
	TRAF5	-0.15	0.06	0.999	-0.33	0.01	0.999
	TRAF6	0.17	0.13	0.999	0.33	0.23	0.999
	TRAF7	0.13	0.08	0.999	-0.01	0.04	0.999
	TXN	-0.22	0.15	0.999	-0.47	0.11	0.999
	UBA52	0.00	0.06	0.999	-0.36	0.02	0.999
	UBC	0.16	0.08	0.999	0.13	0.01	0.999
	USP2	0.34	0.40	0.999	0.25	0.13	0.999
	USP21	0.15	0.06	0.999	0.38	0.01	0.999
	USP4	-0.01	0.07	0.999	0.23	0.03	0.999
	XIAP	-0.23	0.06	0.999	0.04	0.04	0.999
