# Supplementary material for: The actin nucleation factors JMY and WHAMM enable a rapid Arp2/3 complex-mediated intrinsic pathway of apoptosis
Source: PLoS Genet. 2021 Apr 19;17(4):e1009512. doi: 10.1371/journal.pgen.1009512 (PMC8084344; doi:10.1371/journal.pgen.1009512)
Supplement: Attachment — Submitted filename: King_Coverletter+Response.pdf [file pgen.1009512.s018.pdf]

UCONN
UNIVERSITY OF CONNECTICUT

DEPARTMENT OF MOLECULAR AND CELL BIOLOGY
INSTITUTE FOR SYSTEMS GENOMICS

KENNETH G CAMPELLONE, PHD
ASSOCIATE PROFESSOR
67 NORTH EAGLEVILLE RD - RM 216
STORRS, CT 06269-3197

PHONE: (860) 486-3326 LAB: (860) 486-2497
EMAIL: KENNETH.CAMPELLONE@UCONN.EDU
WEB: HTTPS://CAMPELLONELAB.UCONN.EDU
TWITTER: @CAMPELLONELAB

January 6, 2021

Dear Irene and *PLOS Genetics* Editors,

Thank you for your evaluation of our paper entitled "***The actin nucleation factors JMY and WHAMM enable a rapid p53-dependent pathway of apoptosis***" by King et al. for consideration for publication in *PLOS Genetics*. We appreciate the reviewers' constructive comments. Based on their critiques, we are submitting a revised version here entitled "***The actin nucleation factors JMY and WHAMM enable a rapid p53-dependent and Arp2/3 complex-mediated pathway of apoptosis***". As you will see, we have added some exciting new data showing that the Arp2/3 complex and C-terminal motifs in JMY are crucial for apoptosis. JMY appears to play important roles at multiple steps of DNA damage-induced apoptosis, including during the transient assembly of actin-rich cytoplasmic territories containing clusters of cytochrome *c* and cleaved caspase-3.

Due to the addition of more transcriptomic data and protein expression constructs, we have added an additional contributing author, Alyssa M Coulter. We uploaded a 'clean' manuscript text and one with revisions highlighted in yellow to the Editorial Manager. Our point-by-point responses to the reviewers' specific comments are attached below.

Thank you for considering our revised manuscript for publication.

Sincerely,

Ken Campellone
kenneth.campellone@uconn.edu

Dear Ken,

Thank you very much for submitting your Research Article entitled 'The actin nucleation factors JMY and WHAMM enable a rapid p53-dependent pathway of apoptosis' to PLOS Genetics. Our apologies for the delay in reaching a decision, which was due to difficulty in securing appropriate reviewers.

The manuscript has now been fully evaluated by three independent peer reviewers. As you will see, the reviewers appreciated the attention to an important problem, but raised some substantial concerns about the current manuscript, including some of the experimental design and some of the data. Based on the reviews, we will not be able to accept this version of the manuscript, but we would be willing to review again a much-revised version. We cannot, of course, promise publication at that time.

Should you decide to revise the manuscript for further consideration here, your revisions should address the specific points made by each reviewer. We will also require a detailed list of your responses to the review comments and a description of the changes you have made in the manuscript.

If you decide to revise the manuscript for further consideration at PLOS Genetics, please aim to resubmit within the next 60 days, unless it will take extra time to address the concerns of the reviewers, in which case we would appreciate an expected resubmission date by email to plosgenetics@plos.org.

You can use this link to log into the system when you are ready to submit a revised version, having first consulted our Submission Checklist.

To enhance the reproducibility of your results, we recommend that you deposit your laboratory protocols in protocols.io, where a protocol can be assigned its own identifier (DOI) such that it can be cited independently in the future. For instructions see our guidelines.

Please be aware that our data availability policy requires that all numerical data underlying graphs or summary statistics are included with the submission, and you will need to provide this upon resubmission if not already present. In addition, we do not permit the inclusion of phrases such as "data not shown" or "unpublished results" in manuscripts. All points should be backed up by data provided with the submission.

While revising your submission, please upload your figure files to the Preflight Analysis and Conversion Engine (PACE) digital diagnostic tool. PACE helps ensure that figures meet PLOS requirements. To use PACE, you must first register as a user. Then, login and navigate to the UPLOAD tab, where you will find detailed instructions on how to use the tool. If you encounter any issues or have any questions when using PACE, please email us at figures@plos.org.

PLOS has incorporated Similarity Check, powered by iThenticate, into its journal-wide submission system in order to screen submitted content for originality before publication. Each PLOS journal undertakes screening on a proportion of submitted articles. You will be contacted if needed following the screening process.

To resubmit, use the link below and 'Revise Submission' in the 'Submissions Needing Revision' folder.

We are sorry that we cannot be more positive about your manuscript at this stage. Please do not hesitate to contact us if you have any concerns or questions.

Yours sincerely,

Irene Chiolo
Guest Editor
PLOS Genetics

Gregory Barsh
Editor-in-Chief
PLOS Genetics

Reviewer's Responses to Questions

Comments to the Authors:
Please note here if the review is uploaded as an attachment.

Reviewer #1: The actin-nucleation factors JMY and WHAMM enable a rapid p53-dependent pathway of apoptosis

The purpose of this manuscript is to examine potential roles for nucleation promoting factors (NPFs) in programmed cell death. Utilizing Hap1 cells that were genetically depleted of different WASP-family proteins, the authors found that WHAMM and JMY are critical for apoptosis, regulating early steps of cytochrome c release from mitochondria and the caspase-cleavage cascade. The small GTPase RhoD was found to be upregulated in JMY KO cells, and loss of RhoD in Hap1 cells enhanced apoptotic responses to DNA damage, suggesting that RhoD normally promotes cell survival and counterbalances the pro-apoptotic function of JMY.

Overall, this study expands our understanding of apoptosis by identifying two new players in programmed cell death, suggesting a direct link between the actin cytoskeleton and the intrinsic apoptotic pathway. The experiments are thoughtful and straightforward, and direct conclusions are supported by the data. However, the main function of NPFs is the activation of the actin nucleator Arp2/3-complex, the relevance of which has not been addressed in this study. The questions, whether the regulation of apoptosis by WHAMM and JMY is actin-dependent or -independent, and whether both NPFs act in concert in the same pathway or function separately, have been left unanswered. Thus, while I think the manuscript is worthy of publication, a few issues, listed below in more detail, should be addressed beforehand.

Major comments:

1. This study has not addressed whether JMY and WHAMM have specific or redundant functions in apoptosis. Thus, it would be useful to subject JMY/WHAMM double KO cells to Etoposide-treatment and determine the rate of apoptosis in such a cell line. I understand that generating a double KO cell line is time-consuming. Therefore, the authors could alternatively employ one of their JMY siRNAs in WHAMM KO cells.
-- We agree. We now show that WHAMM/JMY DKO cells undergo more apoptosis than the WHAMM KO and the same amount as JMY KO cells. We also performed the suggested siRNA experiments, with similar findings. These new data appear in Fig 4. Collectively, our data support the idea that JMY and WHAMM perform non-redundant functions in the same intrinsic death pathway.

2. Another open question is whether the apoptotic function of JMY and WHAMM is Arp2/3-complex/actin-dependent. I suggest performing RNA-mediated silencing of Arp2 or Arp3 (leading to the loss of the entire complex) and analyzing the rate of apoptosis in these cells. The result might reveal a role for Arp2/3-complex in DNA damage-induced apoptosis, indicating an Arp2/3-dependent role for JMY and WHAMM in this process.
--We agree. We now show that cells treated with siRNAs that deplete the Arp2/3 complex, or with a small molecule inhibitor of the Arp2/3 complex, undergo less apoptosis. Moreover, targeting of the Arp2/3 complex in WHAMM KO cells, but not JMY KO cells, results in less apoptosis. These new data appear in Fig 8. Collectively, our data demonstrate that JMY, WHAMM, and Arp2/3 functions are all required for efficient intrinsic apoptosis. These results (along with p53 targeting in Fig 5 and S6) further indicate that apoptosis levels in JMY KO cells are nearly equivalent to those in p53-deficient cells, and imply that the residual apoptosis levels

in WHAMM KO cells are p53-, JMY-, and Arp2/3-mediated.

3. I am aware that rescue experiments are not trivial in general, however, it would be interesting to re-express JMY wt versus a JMY-WCA mutant in JMY KO cells and test for efficiency of apoptosis. Along with point 2., this could shed light on the relevance of JMY's function as an Arp2/3-complex activator for apoptosis. Furthermore, it would be a good control experiment to show that JMY wt is able to rescue the observed apoptosis phenotype.
-- We agree. We now show that apoptosis (measured by caspase-3 activation) in JMY KO cells could be rescued with full-length murine JMY, but not JMY mutants lacking the Arp2/3-binding CA motif or the actin-binding WWW segment. These new data appear in Fig 8. Thus, combined with experiments addressing point #2 above, our data indicate that JMY-mediated apoptosis requires its ability to activate the Arp2/3 complex.

4. I assume that the authors had problems to find a functional/specific RhoD antibody, which is why they used RT-PCR to examine the transcript levels of this protein in cells. Nonetheless, it would be of major advantage for the manuscript to show the increase of endogenous RhoD in JMY KO cells at the protein expression level. Given that mRNA levels of RhoD are undetectable in control Hap1 cells by RT-PCR, the determination of RhoD protein levels is even more relevant. Furthermore, the authors have not analyzed RhoD expression in WHAMM KO cells, although it would be interesting to analyze whether the increased expression of RhoD is a general cell response in order to cope with apoptosis defects, especially in regard to WHAMM being an interactor of RhoD.
--Yes, we have tried several RhoD antibodies, but none have been able to detect endogenous RhoD in HAP1 cells, eHAP cells, or their derivatives. We agree with the idea that RhoD upregulation might be a general cellular response to any of a number of types of stress. But our unpublished RNAseq data indicate that *RHOD* is not turned on in WHAMM KO cells (FPKM = 11.06 in eHAP, 8.60 in WHAMM KO; see comment to Reviewer 2 below for RT-PCRs). We believe that cells can "adjust" to the loss of JMY and/or WHAMM in several ways in order to maintain homeostasis. As further evidence of this, we have added data to Fig 6 showing that *RHOD* is turned on in only 2 of the 3 JMY KO cell lines – presumably the third line has found different (epigenetic) ways to cope with *JMY* inactivation. Finally, we expressed GFP-RhoD to show that the protein can be recruited around JMY and cytochrome *c*-containing clusters in apoptotic cells. These data are presented in Fig 10.

Minor comments:

Fig 1A: I would prefer that the authors show representative images for all KO cell lines, even the ones that had no effect on apoptosis.
--We have added the requested images to Fig S1.

Fig 4E and F: The marker band for 'Casp-3 cleaved' is depicted as 20 kDa in E and 25 kDa in F.
--Thanks for catching that, we have corrected the labels.

Fig 6B: This is a rather modest effect for siTP53, which indicates that Etoposide-induced apoptosis is not entirely driven by p53. Is there a p53-independent apoptotic pathway? And can the authors exclude that JMY is also functioning in p53-independent apoptotic pathways? Comment on that.
--When p53 depletions are extrapolated to zero protein remaining, the knockdown cells would be predicted to undergo apoptosis at approximately 10-12% (see Y-axis in Fig 5). This closely matches the 8-12% apoptosis values observed in JMY KO cells or those extrapolated from JMY siRNA-treated cells, leading to the conclusion that the apoptosis occurring in this remaining 10%

of cells is independent of p53 and JMY.

Fig 6F and G: Why were only control Hap1 cells examined for nuclear translocation of p53? Although the figure title says that 'JMY is not required for nuclear accumulation of p53', JMY KO cells are not shown in part F and G.
--Those data were missing as a consequence of the Covid shutdown, but we have now added the images showing that p53 is abundant in JMY KO nuclei in Fig S7.

Fig 7A: What is (RU)? I assume the authors mean AU?
--Relative Units.

S1Table: The BRK1 KO cell line is referred to as WAVE complex KO, which I have a hard time to believe from what is known about the WAVE complex. Deletion of components, such as Sra1, Nap1 or WAVE have been shown to significantly affect the expression of the entire complex. However, the small component BRK1 (~10 kDa) most likely does not affect the expression of the remaining components. Do the authors have data to confirm that the expression of the entire complex is affected by loss of BRK1?
--We targeted *BRK1* based on the findings of (Linkner et al., PLOS ONE, 2011) and references cited therein. We previously found that siRNAs targeting *Brk1* results in substantial decreases in the expression and function of WAVE1 and WAVE2 in mouse cells (Kabrawala et al., PLOS Genetics, 2020). For the current study, our immunoblotting indicates that relatively little WAVE1 (<10% of that in parental HAP1 cells) and WAVE2 (<20% of that in parental HAP1 cells) is present in the BRK1 (WAVE complex) KO cells. Moreover, the BRK1 (WAVE complex) KO cells are noticeably smaller/rounder than the parental HAP1 and individual WAVE KO cells, phenotypically demonstrating that the *BRK1* mutation has a major effect on WAVE complex function. These data are now presented in Fig S1.

Line 525: remove 'in'
--Corrected.

Reviewer #2: The manuscript here reports a role of the actin nucleation factors JMY and WHAMM in etoposide-induced apoptosis. Using knockdown and knockout approach, it was shown that deletion/depletion of JMY and WHAMM, as well as depletion of p53, reduced the etoposide-induced apoptosis. RNA-seq analysis revealed increased levels of RhoD in JMY knockout cells. Furthermore, knockout of RhoD sensitized cells to etoposide treatment, suggesting it may be the pro-survival factor in the JMY-deleted cells. In all, despite that the results in general supported a role of JMY and WHAMM in etoposide-induced apoptosis, the physical and functional links with either p53 or RhoD were not established. More convincing evidence is required.

1. Fig. 2 and 3 are redundant. Experiments with siRNA knockdown (Fig. 3) can be moved to supplementary data.
--The previous Fig 2 (permanent KO) and Fig 3 (transient KD) address two different biological questions – what is apoptosis like in cells completely lacking the given protein for many generations vs what is apoptosis like in cells immediately after significant depletion of that protein (but prior to the selective pressures of extended culturing). In our case, both the permanent and temporary loss of JMY resulted in similar phenotypes (less apoptosis). The knockdown experiments have been moved to Fig 5 to provide direct comparisons to p53 knockdowns. Transient depletion of JMY or p53 gave notably parallel results.

2. There are many post-translational modification sites in p53 that may have differential impacts on p53-targeted promoters. For apoptosis, pS46 may be more relevant than pS15. The involvement of pS46 should be investigated (Fig. 6). Furthermore, induction of apoptosis-related genes, such as Bax and PUMA, should be compared.
--We have now assessed phospho-S46 levels by immunoblotting and show that they are indistinguishable in HAP1 and JMY KO cells in the current Fig S7. Further, RNAseq comparing HAP1 and JMY KO cells did not reveal any statistically significant changes in *BAX* or *PUMA* transcript levels at steady state, and new RNAseq and RT-PCR data following etoposide treatment reveals that *BAX* and *PUMA* transcript levels increased to a similar extent in HAP1 and JMY KO cells, as shown in Figs 6 and S10. We do not have any indications that differences in *BAX* or *PUMA* gene expression can account for the significant defects in apoptosis that we observe in the absence of JMY.

3. The authors suggested that cell arrest may have occurred in etoposide-treated JMY KO cells (Fig. 7). Flow cytometry analysis is needed to verify this point. Induction of CDKN1A can hardly address this point since HAP1 cells undergo apoptosis despite similar amount of CDKN1A induction.
--We indicated that the cells stopped proliferating in Fig 7 (current Fig S8). This was apparent from simply counting the cells, as well as quantifying AnnV positivity and negativity and numbers of nuclei in the different populations. Our previous depiction of cell numbers in the Fig was somewhat confusing, so we have clarified this quantification in the current version. Both parental and JMY KO cells stop proliferating and upregulate the cell cycle inhibitor *CDKN1A* (p21) to a similar extent, as shown in an updated Figs S8-S10. The point here is that both sets of cells undergo a proliferative arrest, consistent with some of the known functions of p53 and p21, but that the KO cells have a specific defect in death, leading us to conclude that JMY functions at a different/later step in apoptosis.

4. RNA-seq appeared to be conducted with samples collected from untreated cells. As DNA damage response is being investigated, it may be more appropriate to compare untreated and etoposide-treated HAP1 and JMY KO cells.
--We agree. We have added RNAseq data comparing etoposide-treated HAP1 and JMY KO cells to new Figs 6, S9, S10. In the revised paper, we describe the fact that both sets of cells up- or down-regulate the same genes to similar degrees. The only major differences between the two cell types were in the upregulation of distinct cytokines (*CCL20* and *IL8* vs *TNF*). We followed this up with a few targeted RT-PCRs on specific pro- or anti-apoptotic factors. As mentioned above, the two cell populations showed similarities in *BAX*, *PUMA,* and *BCL2* levels, suggesting that differences in the regulation of these factors is unlikely to be the primary driver of the higher levels of death in wild type vs JMY KO cells.

5. RT-PCR products shown in Fig. 8C and 8E were mostly overly amplified (except RhoD and WHAMM), which could easily mask the difference. Reduced amplification cycle should be tested, or alternatively, qPCR can be used.
--We added new panels to emphasize that *RAB1* and *ACTIN* levels do not differ between the cell types (all consistent with the RNAseq data as well). These data are shown in Fig 6.

6. Although RhoD expression is elevated in JMY KO cells, data presented did not support its involvement in the reduced apoptosis seen in JMY o WHAMM KO cells. Depletion of RhoD did not exacerbate etoposide-induced apoptosis in JMY KO cells, suggesting they may not function in the same axis (Fig. 9C). As for its role in WHAMM related apoptotic pathway, it may be more appropriate to use the WHAMM KO eHAP cells (Fig. 9E-9I).

--We think that RhoD is a general pro-survival protein that is upregulated in response to stress, as we showed that knocking out *RHOD* causes increases in necrosis in the absence of JMY and increases in apoptosis and necrosis in the presence of JMY (current Figs 7, S11). Depletion of *RHOD* did not increase apoptosis in JMY KO cells, because JMY KO cells (similar to p53-depleted cells) are fundamentally defective in apoptosis (Fig 5). Our data presented in a new Fig 10 indicates that in the absence of *RHOD*, the transient assembly of JMY and cytochrome c in cytoplasmic clusters is more frequent. GFP-RhoD localizes around these clusters, and the nucleotide-bound state of RhoD affects their morphogenesis. As the reviewer suggested, we knocked down *RHOD* in the WHAMM KO cells. These cells have only partial apoptotic deficiencies, and as shown here below, *RHOD* silencing in this background can increase apoptosis and necrosis.

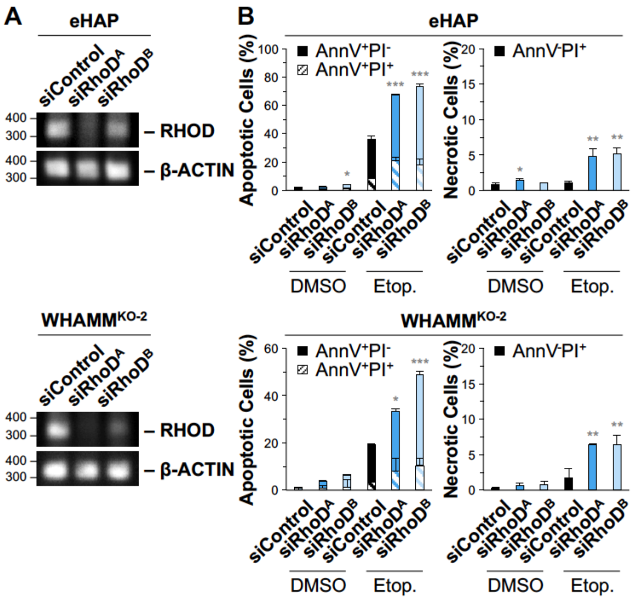

7. Fig. 10 nicely showed that RhoD is a pro-survival factor under etoposide treatment in eHAP1 cells. However, it also has not addressed the relationship with JMY or WHAMM. It can be presented as a supplementary data for Fig. 9.
--We think that the pro-survival function of RhoD is important enough to present in the current Fig 7, and the relationship of RhoD to JMY localization is tied more closely together in the current Fig 10. Other RhoD data have been moved to Fig S11.

Minor points:
1. At some points, JMY KO cells were referred as JMY mutant cells, which could be easily confused with point mutant.
--The identities of the frameshift-causing genetic mutations (2bp, 10bp, and 17bp) in *JMY* are shown in Table S1 and Fig S3. These mutations do not cause targeted amino acid substitutions in JMY, yet the knockout cell lines would still be considered mutant cell lines. Targeted small deletions in GFP-JMY rescue constructs (JMYΔCA and JMYΔWWW) are specifically defined in the revised manuscript.

2. Discussion is a bit too long.
--Acknowledged.

Reviewer #3: King and colleagues report that two proteins known to function in actin assembly have roles in promoting apoptosis. Modulators of actin dynamics and actin itself are now known to medicate apoptosis, leading to the view that actin cytoskeleton is a biosensor that integrates internal and external apoptotic stimuli. But these studies typically focus on proteins that depolymerize or cross-link actin. The current study is on proteins that promote actin polymerization, thus filling a knowledge gap. While the study is suitable for PLoS Genetics in principle, problems with the experimental design and some of the data prevent me from recommending publication. The data that JMY- or WHAMM-depleted cells show reduced caspase activation and reduced apoptosis after etoposide treatment (Figs 1-4) are convincing, as are the (negative) data on the role of JMY in p53 activation (Fig. 6). Problematic data/conclusions are:

1. Conclusions made based on data from different cell lines. For example, the effect JMY depletion was studied in Hap1 (nearly haploid) but the effect of WHAMM depletion was studied in eHap (fully haploid, derived from Hap1 by CRISPR). These cell lines behave differently as seen later in RhoD experiments, yet the authors make conclusions across cell lines. For example, JMY was concluded to be more important for apoptosis than WHAMM (line 150 and models in Fig. 10). To make such a conclusion, knocked down phenotypes must be compared in the same cell line.
--In the revised paper, we confirmed that HAP1 and eHAP cells behave identically in assays of apoptosis. This is despite the fact that the eHAP cells express more *RHOD*. We have performed knockdown assays in both HAP1 and eHAP cells, which differ only in the diploidy of ~300 genes (one of the genes is *WHAMM*) from chromosome 15. The respective apoptotic phenotypes upon siRNA targeting of JMY, WHAMM, Arp2/3, or TP53 are indistinguishable in HAP1 vs eHAP cells (Figs 4, 5, 8, S5, S6). A role for JMY was also confirmed in U2OS and HeLa cells (Fig S6).

2. If proteins being tested are in a complex, why did depletion of just one or two members showed a phenotype? The authors should confirm the success of the knockout for genes that did not give a phenotype, by Western blotting like they did for genes that did give a phenotype.
--WASP-family proteins are not in complexes with one another. They are in separate complexes that activate the Arp2/3 complex. Nevertheless, we have added immunoblots for the other knockouts in a new Fig S1.

3. Cytochrome C data in Fig. 5 is not convincing. I cannot see the difference between Hap1 and JMY ko (drug-treated) in A nor between eHap and WHAMM ko (drug treated cells) in B, yet quantifications in C and D show large differences. How exactly are the images quantified? It seems to be a subjective YES/NO call for whether cytochrome C is mitochondrial or cytoplasmic but how is that call being made? Along the same lines, Figure S3D reports the number of gamma-H2AX foci, but based on the images in B, I do not see how the authors can quantify foci number, especially in drug-treated cells. It is very hard to see discrete foci. The Western blot data in S3A is convincing, however.
--To further clarify the mitochondrial versus diffuse cytoplasmic localization of cytochrome c, we have provided larger magnifications in Fig 2. To emphasize the differences in mitochondrial versus diffuse cytochrome c, we have also added fluorescence plot-profiles in a new Fig 2C-D. These also highlight the yes/no scoring system used in panel E. To address the quantification of H2AX foci, we have added a magnification in Fig S4. Upon etoposide-induced damage, the cells have a lot of H2AX foci that ImageJ can resolve and count.

4. Figure 7 is perplexing. The authors set up this figure by saying that inhibition of cell

proliferation is an early response to DNA damage (line 234), yet when they address this experimentally, they are not looking at the early pause in the cell cycle (presumably checkpoint-mediated) but what happens over ~2 days after the drug has been washed out. Here, they see an ~50% increase in Hap1 cell number at the 6h time point compared to the 0h time point, which does not make sense because at 5uM of drug used in this experiment, 40% of Hap1 cells should be dead (Fig. 2C) and the rest arrested in the cell cycle by checkpoints. Over the next 48hr, the relative number of Hap1 cells stayed the same but % that is Annexin 5+ increased to reach 100%. Why does the cell number stay the same when larger and larger fractions of them are dying? Are they reaching a steady state because proliferation is off-set by death? Are dead cells included in the cell count? Are cells showing signs of apoptosis but not really dead? It is important to understand what is happening in Hap1 controls in this experiment so that the data for JMY knock down cells can be interpreted. If conclusions are to be made about 'cell cycle arrest', cell cycle status should be examined directly.
-- Our previous depiction of cell numbers in the Fig was somewhat confusing, so we have clarified this quantification in the current version. We point out the AnnV-positive versus AnnV-negative and total cell scoring systems in a revised Fig S8. DMSO-treated cells continue to divide so the number of total cells continues to rise (e.g. lines with positive slopes in panel B). Etoposide-treated cells stop dividing (flat lines in B). Among those non-dividing Etoposide-treated cells, the fraction that are apoptotic (AnnV-positive) increases from 6-48h, while the fraction that are non-apoptotic (AnnV-negative) decreases – the total number of cells (AnnV-positive plus AnnV-negative) stays roughly the same (panels A-C). The JMY KO cell numbers parallel those of the HAP1 cells, but remain mostly in the AnnV-negative category. Because we haven't explicitly addressed the stage(s) of the cell cycle in which the WT and KO cells arrest, we now refer to this as a proliferation arrest rather than a cell cycle arrest in the current figures.

5. While RhoD RNA is induced in JMY ko Hap1 cells, depletion of it makes little difference to apoptosis. So, the reason for reduced apoptosis in JMY ko cells is still unknown. Depletion of RhoD in eHap cells did increase apoptosis. Why don't the authors study the effect of JMY depletion on apoptosis and RhoD expression in eHap cells? They went as far as confirming the role of JMY in Hela and U2OS cells but not in eHap. If the results in eHap cells are positive, then they can ask if increased RhoD is the reason JMY ko cells show increased apoptosis. Again, because the requirement for different genes are shown in different cell lines, the conclusions in Fig. 10F are not justified by the data. In fact, this major problem with the experimental design prevents the authors from making impactful conclusions.
--As mentioned above, we have added JMY knockdown experiments in eHAP cells in the current Fig 4. As expected, JMY depletion also reduced apoptosis levels (assessed via AnnV staining) in eHAP cells. The HAP1 and eHAP cells only differ genetically by a duplicated ~300 gene region from chromosome 15, and the two cell lines have been phenotypically equivalent in all functional assays that we have performed.

Have all data underlying the figures and results presented in the manuscript been provided?

Large-scale datasets should be made available via a public repository as described in the *PLOS Genetics* data availability policy, and numerical data that underlies graphs or summary statistics should be provided in spreadsheet form as supporting information.

Reviewer #1: Yes

Reviewer #2: Yes

Reviewer #3: Yes
