# Supplementary material for: The actin nucleation factors JMY and WHAMM enable a rapid Arp2/3 complex-mediated intrinsic pathway of apoptosis
Source: PLoS Genet. 2021 Apr 19;17(4):e1009512. doi: 10.1371/journal.pgen.1009512 (PMC8084344; doi:10.1371/journal.pgen.1009512)
Supplement: Attachment — Submitted filename: King_Coverletter+Response2b.pdf [file pgen.1009512.s019.pdf]

UCONN
UNIVERSITY OF CONNECTICUT

DEPARTMENT OF MOLECULAR AND CELL BIOLOGY
INSTITUTE FOR SYSTEMS GENOMICS

KENNETH G CAMPELLONE, PHD
ASSOCIATE PROFESSOR
67 NORTH EAGLEVILLE RD - RM 216
STORRS, CT 06269-3197

PHONE: (860) 486-3326 LAB: (860) 486-2497
EMAIL: KENNETH.CAMPELLONE@UCONN.EDU
WEB: HTTPS://CAMPELLONELAB.UCONN.EDU
TWITTER: @CAMPELLONELAB

March 10, 2021

Dear Irene and *PLOS Genetics* Editors,

Thank you for your evaluation of our paper entitled "***The actin nucleation factors JMY and WHAMM enable a rapid p53-dependent and Arp2/3 complex-mediated pathway of apoptosis***" by King et al. for consideration for publication in *PLOS Genetics*. We appreciate the constructive comments and are submitting a revised version here entitled "***The actin nucleation factors JMY and WHAMM enable a rapid Arp2/3 complex-mediated intrinsic pathway of apoptosis***". We have removed "p53-dependent" from the title to avoid any inferences that direct physical interactions between JMY/WHAMM and p53 are modulating the cytoplasmic aspects of this pathway. We have also provided clearer image magnifications to address previous ambiguity in our JMY-cytochrome *c* localization studies, and made several text modifications.

We uploaded a 'clean' manuscript text and one with revisions highlighted in cyan to the Editorial Manager. Our point-by-point responses to the editors' and reviewers' specific comments are attached below and shown in blue.

Thank you for considering our revised manuscript for publication.

Sincerely,

Ken Campellone

kenneth.campellone@uconn.edu

Dear Dr. Campellone,

Thank you very much for submitting your Research Article entitled 'The actin nucleation factors JMY and WHAMM enable a rapid p53-dependent and Arp2/3 complex-mediated pathway of apoptosis' to PLOS Genetics.
The manuscript was fully evaluated at the editorial level and by independent peer reviewers. The reviewers appreciated the attention to an important topic but identified some additional concerns that we ask you address in a revised manuscript.

We therefore ask you to modify the manuscript according to the review recommendations. Your revisions should address the specific points made by each reviewer. We believe that the remaining concerns can be addressed to our satisfaction by text changes, and will not require the addition of new data. We recommend a particular attention to the following points:

- With the added data, it is becoming more clear that the pathway controlled by JMY and WHAMM is independent of and is different from that by p53, yet the title still indicates that p53 and JMY/WHAMM work together in etoposide-induced apoptosis.
--As mentioned above, we have removed “p53-dependent” from the title and incorporated the term “intrinsic”. The reviewer is correct in that we do not believe the primary functions of JMY/WHAMM in the intrinsic apoptotic pathway involve direct physical interactions with p53 in the cytoplasm (if that is what they mean by ‘work together’). However, as explained below, the pathway remains p53-dependent: JMY and WHAMM (like cyto *c* and the caspases) simply act downstream (not independent) of p53’s earlier nuclear functions.

- The RhoD DN data in the new Fig. 10 is also conflicting as it decreased the JMY puncta.
--We do not see these data as conflicting. RhoD can exist in GTP- or GDP-bound forms. As described in our response below, the WT form of RhoD, which is a predominantly GTP-bound species (Aspenstrom, 2020), surrounds the JMY puncta but does not interfere with their formation (Fig 10C-E). In contrast, the GDP-bound mutant increases JMY retention in the nucleus and *does* interfere with the formation of JMY puncta (Fig 10C-E). So when endogenous RhoD is deleted (as in Fig 7 or 10B), the inhibitory GDP-bound form is lost and cells die more frequently. Thus, our observation that RhoD-GDP can ‘apply the brakes’ to the intrinsic apoptotic pathway ties together the knockout and overexpression phenotypes.

- The authors now provide new data in Fig. 8F, 9 showing that JMY sometimes appeared in bright, juxtanuclear clusters that seemed to overlap with cleaved caspase-3, cyto c and F-actin staining. This association of JMY-positive structures with cyto c and F-actin provides an interesting new data set for the overall conclusions in the manuscript. However, while the association of JMY with a subset of cyto c puncta is somewhat visible in Fig. 9C and 9G, there is clearly no co-localization of JMY and cyto c in Fig. 9E, on the contrary the signals are mutually exclusive. Similarly, in Fig. S12 cyto c and JMY are not overlapping in those clusters, also actin is not directly associating with either one of them. Therefore, I would strongly advice to remove Fig. 9E (and S12A) and overall soften statements about overlap/co-localization of these factors.
-- We agree with Reviewer 1’s assessment, mainly because we did not do a good job of cropping, presenting, and explaining panels 9E, S12A, and S12B. The brightness of mitochondria in the periphery of the magnifications dominated some images, which made the centrally-located puncta poorly visible. In the current version, we re-cropped the 9E and S12B images and replaced the S12A image to better highlight the consistent overlap between JMY and cyto *c* in non-mitochondrial puncta. We have softened several statements in the text and refer to the presence of JMY, cyto *c*, and active caspase-3 within F-actin-rich “territories” rather than saying that they co-localize with F-actin.

In addition we ask that you:
1) Provide a detailed list of your responses to the review comments and a description of the changes you have made in the manuscript.
--Responses and descriptions of changes appear below. (In sum: title change; better cropping of images in Figs 9, S12; minor text changes highlighted in cyan of the uploaded manuscript).

2) Upload a Striking Image with a corresponding caption to accompany your manuscript if one is available (either a new image or an existing one from within your manuscript). If this image is judged to be suitable, it may be featured on our website. Images should ideally be high resolution, eye-catching, single panel square images. For examples, please browse our archive. If your image is from someone other than yourself, please ensure that the artist has read and agreed to the terms and conditions of the Creative Commons Attribution License. Note: we cannot publish copyrighted images.
--Uploaded.

We hope to receive your revised manuscript within the next 30 days. If you anticipate any delay in its return, we would ask you to let us know the expected resubmission date by email to plosgenetics@plos.org.

If present, accompanying reviewer attachments should be included with this email; please notify the journal office if any appear to be missing. They will also be available for download from the link below. You can use this link to log into the system when you are ready to submit a revised version, having first consulted our Submission Checklist.

While revising your submission, please upload your figure files to the Preflight Analysis and Conversion Engine (PACE) digital diagnostic tool. PACE helps ensure that figures meet PLOS requirements. To use PACE, you must first register as a user. Then, login and navigate to the UPLOAD tab, where you will find detailed instructions on how to use the tool. If you encounter any issues or have any questions when using PACE, please email us at figures@plos.org.

Please be aware that our data availability policy requires that all numerical data underlying graphs or summary statistics are included with the submission, and you will need to provide this upon resubmission if not already present. In addition, we do not permit the inclusion of phrases such as "data not shown" or "unpublished results" in manuscripts. All points should be backed up by data provided with the submission.
Please review your reference list to ensure that it is complete and correct. If you have cited papers that have been retracted, please include the rationale for doing so in the manuscript text, or remove these references and replace them with relevant current references. Any changes to the reference list should be mentioned in the rebuttal letter that accompanies your revised manuscript.

PLOS has incorporated Similarity Check, powered by iThenticate, into its journal-wide submission system in order to screen submitted content for originality before publication. Each PLOS journal undertakes screening on a proportion of submitted articles. You will be contacted if needed following the screening process.

To resubmit, you will need to go to the link below and 'Revise Submission' in the 'Submissions Needing Revision' folder.

Please let us know if you have any questions while making these revisions.

Yours sincerely,

Irene Chiolo
Guest Editor
PLOS Genetics

Gregory Barsh
Editor-in-Chief
PLOS Genetics

Reviewer's Responses to Questions

Comments to the Authors:

Please note here if the review is uploaded as an attachment.

Reviewer #1: The authors showed that both loss of JMY and WHAMM decreased p53-dependent apoptosis in cells but did not address the relationship of both NPFs in this pathway. Here, in the revised version they included evidence for JMY and WHAMM acting non-redundantly in the same apoptotic pathway. Interestingly, the additional KO of JMY in WHAMM KO cells caused a decrease in Etoposide-induced apoptosis to the same extent as JMY KO alone, indicating that JMY is more potent and their effects are non-additive. Furthermore, the authors compared permanent loss of JMY/WHAMM with transient siRNA-mediated depletion of both factors with similar results. This is an important control experiment and I agree with the authors response to reviewer #2, that permanent, long-term and instant, transient protein removal might have diverse effects and needed to be tested.

New Fig. 8 adds an important point to the manuscript, namely that JMY/WHAMMs roles in apoptosis are Arp2/3- and thus actin-dependent. This conclusion might seem trivial as these proteins are first and foremost nucleation-promoting factors. However, since JMY also has functions in transcriptional regulation, it was important to clarify if its particular role in apoptosis is Arp2/3 complex-dependent or -independent.

I also appreciate the addition of new Fig. 10, which shows that expressed RhoD can be found around JMY/cyto c containing clusters, providing more evidence for an implication of RhoD in this pathway, besides the elevated expression found in 2 of the 3 JMY KO clones.

Taken together, the authors have sufficiently addressed all my comments and strengthened the manuscript with new findings. Thus, while I generally support publication, I would like to point out a few things regarding the new data.

The authors now provide new data in Fig. 8F, 9 showing that JMY sometimes appeared in bright, juxtanuclear clusters that seemed to overlap with cleaved caspase-3, cyto c and F-actin staining. This association of JMY-positive structures with cyto c and F-actin provides an interesting new data set for the overall conclusions in the manuscript. However, while the association of JMY with a subset of cyto c puncta is somewhat visible in Fig. 9C and 9G, there is clearly no co-localization of JMY and cyto c in Fig. 9E, on the contrary the signals are mutually exclusive. Similarly, in Fig. S12 cyto c and JMY are not overlapping in those clusters, also actin is not directly associating with either one of them. Therefore, I would strongly advice to remove Fig. 9E (and S12A) and overall soften statements about overlap/co-localization of these factors.

-- We agree with Reviewer 1's assessment, mainly because we did not do a good job of cropping, presenting, and explaining panels 9E, S12A, and S12B. The brightness of mitochondria in the periphery of the magnifications dominated some images, which made the centrally-located puncta poorly visible. In the current version, we re-cropped the 9E and S12B images and replaced the S12A image to better highlight the overlap between JMY and cyto *c* in non-mitochondrial puncta. (Imaging the transient cytoplasmic clouds of cleaved-DEVD for 9E is incredibly challenging.) We have also softened several statements in the text and refer more consistently to the presence of JMY, cyto *c*, and active caspase-3 in F-actin-rich "territories" (rather than saying that they co-localize with F-actin). These zones of F-actin that encompass the apoptotic factors are much brighter than the F-actin in the rest of the cell, which is best seen in the lower magnification panels. Improved images appear in Fig 9E and S12A,B. Text changes appear in lines 636-638, 696-705, 730-731, 1495-1500.

Reviewer #2: In this revised manuscript, additional data have been added and some concerns were addressed. The authors also added the Arp2/3 part to provide relevant mechanistic explanation. These old and new data, adding together, support the involvement of p53, JMY, WHAMM, RHOD, and Arp2/3 in regulating etoposide-induced apoptosis but unfortunately do not offer a coherent picture regarding the functional or physical connection between them. There are also issues related to interpretation and experimental designs. The manuscript should be restructured and may be reorganized to present these significant amounts of data. Examples of relevant issues are:

1. Although the manuscript started with examination of JMY and WHAMM, the majority body of data, experimental designs, and interpretations seemed to focus on JMY. This is further complicated by some of the results that suggest the effect of JMY and WHAMM may regulate apoptosis differently.
--Yes, we included WHAMM but focused on JMY, the nucleation factor with the more influential role phenotypically. We ended up with >110 data panels. If we performed every JMY experiment with WHAMM, the manuscript size would be even less manageable.

2. With the added data, it is becoming more clear that the pathway controlled by JMY and WHAMM is independent of and is different from that by p53, yet the title still indicates that p53 and JMY/WHAMM work together in etoposide-induced apoptosis.
--As mentioned above, we removed p53 from the title. But we disagree with this Reviewer's interpretation that the JMY/WHAMM-mediated effects are independent of p53. The actin assembly factors simply function downstream (not independent) of the nuclear activities of p53 (just as cyto *c*, apoptosome components, and caspases do). We agree with the reviewer that p53 may not physically interact with JMY/WHAMM in the cytoplasm, and we do not mean to imply that JMY/WHAMM "work together" directly with p53, just that they are all part of the same intrinsic death pathway. HAP1 and JMY KO cells both exhibit all the widely-accepted features of DNA damage-induced p53-dependent intrinsic apoptosis: p53 is upregulated (S7A,C), accumulates in the nucleus (S7B), is phosphorylated and acetylated (S7A), the p53 target gene *CDKN1A* is upregulated (S8D; S9B; S10), the protein product p21 is expressed and found in the nucleus (S8E-F), the cells arrest (S8A-C), and some pro-apoptotic p53-target genes are upregulated (6D; S10). Moreover, etoposide-induced death in HAP1/eHAP cells is p53-dependent: the apoptosis (AnnV) phenotype of p53-depleted cells closely parallels those of JMY/WHAMM knockdowns or knockouts (Fig 5A-F; S6A-C). If were to deplete cyto *c* or Apaf-1 (an apoptosome component) or caspase-9 or caspase-3 and saw apoptotic defects similar to those following JMY/WHAMM depletion, we would not argue that any of these other factors are p53-independent. They just all operate later in the pathway, downstream of p53's earlier activities.

3. The upregulation of RhoD in JMY KO cells may not convincingly explain the increased survival as it was not among the genes particularly enhanced by the etoposide treatment. The RhoD DN data in the new Fig. 10 is also conflicting as it decreased the JMY puncta. Besides, why wasn't this exp. conducted in RhoD KO cells?
--Correct, we do not claim that the upregulation of *RHOD* explains increased survival following etoposide treatment, and *RHOD* was not further upregulated upon etoposide treatment. JMY KO cells are fundamentally defective in intrinsic apoptosis, similar to p53-depleted cells. We believe RhoD plays a basic pro-survival role in cells and have no evidence to the contrary. For the RhoD transfection data, as mentioned from p.29-32, the experiment was performed in U2OS cells because we were assessing the effects of overexpressing (not rescuing) RhoD protein on the JMY structures in a cell line that is more amenable to transient transfection and imaging.

The data are not conflicting: In cells, RhoD is mostly in the GTP-bound form, but can be switched to the GDP-bound form depending on the presence of GAP proteins in the cell (Aspenstrom, 2020). So when it is expressed at normal levels, cells die at regular frequencies (i.e., Fig 10B), presumably due to a coordinated balance between the GTP- and GDP-bound species. The GTP-bound form surrounds the JMY puncta but does not interfere with their formation (Fig 10C-E). In contrast, the GDP-bound form increases JMY retention in the nucleus and *does* interfere with the formation of JMY puncta (Fig 10C-E). So when RhoD is depleted or deleted (as in Fig 7 or 10B), the inhibitory GDP-bound version is lost and cells die more frequently.

4. Additional evidence on how alteration of actin filament, either its polymerization or organization, affects release of cytochrome c or apoptosis could strengthen the Arp2/3 data.
--Yes, it could. And we hope to continue to investigate how actin and the nucleation machinery affect multiple steps of apoptosis. But we're stopping here with 20+ figures and 110+ data panels.

Reviewer #3: The revised manuscript by King and colleagues addresses my concerns from the first round of review. New data on the role of Arp2/3 in apoptosis strengthens the story. I support the publication of this work.
--Thanks.

Have all data underlying the figures and results presented in the manuscript been provided?
Large-scale datasets should be made available via a public repository as described in the *PLOS Genetics* data availability policy, and numerical data that underlies graphs or summary statistics should be provided in spreadsheet form as supporting information.
Reviewer #1: Yes
Reviewer #2: Yes
Reviewer #3: Yes
